# Comparative Genome-Wide Screening Identifies a Conserved Doxorubicin Repair Network That Is Diploid Specific in *Saccharomyces cerevisiae*

**DOI:** 10.1371/journal.pone.0005830

**Published:** 2009-06-08

**Authors:** Tammy J. Westmoreland, Sajith M. Wickramasekara, Andrew Y. Guo, Alice L. Selim, Tiffany S. Winsor, Arno L. Greenleaf, Kimberly L. Blackwell, John A. Olson, Jeffrey R. Marks, Craig B. Bennett

**Affiliations:** 1 Department of Surgical Sciences, Duke University Medical Center, Durham, North Carolina, United States of America; 2 North Carolina School of Science and Mathematics, Durham, North Carolina, United States of America; 3 Department of Biochemistry, Duke University Medical Center, Durham, North Carolina, United States of America; 4 Department of Medicine, Duke University Medical Center, Durham, North Carolina, United States of America; Texas A&M University, United States of America

## Abstract

The chemotherapeutic doxorubicin (DOX) induces DNA double-strand break (DSB) damage. In order to identify conserved genes that mediate DOX resistance, we screened the *Saccharomyces cerevisiae* diploid deletion collection and identified 376 deletion strains in which exposure to DOX was lethal or severely reduced growth fitness. This diploid screen identified 5-fold more DOX resistance genes than a comparable screen using the isogenic haploid derivative. Since DSB damage is repaired primarily by homologous recombination in yeast, and haploid cells lack an available DNA homolog in G1 and early S phase, this suggests that our diploid screen may have detected the loss of repair functions in G1 or early S phase prior to complete DNA replication. To test this, we compared the relative DOX sensitivity of 30 diploid deletion mutants identified under our screening conditions to their isogenic haploid counterpart, most of which (n = 26) were not detected in the haploid screen. For six mutants (*bem1Δ, ctf4Δ, ctk1Δ, hfi1Δ,nup133Δ, tho2Δ)* DOX-induced lethality was absent or greatly reduced in the haploid as compared to the isogenic diploid derivative. Moreover, unlike WT, all six diploid mutants displayed severe G1/S phase cell cycle progression defects when exposed to DOX and some were significantly enhanced (*ctk1Δ* and *hfi1Δ*) or deficient (*tho2Δ*) for recombination. Using these and other “THO2-like” hypo-recombinogenic, diploid-specific DOX sensitive mutants (*mft1Δ, thp1Δ, thp2Δ*) we utilized known genetic/proteomic interactions to construct an interactive functional genomic network which predicted additional DOX resistance genes not detected in the primary screen. Most (76%) of the DOX resistance genes detected in this diploid yeast screen are evolutionarily conserved suggesting the human orthologs are candidates for mediating DOX resistance by impacting on checkpoint and recombination functions in G1 and/or early S phases.

## Introduction

Doxorubicin (DOX) is a highly effective anthracycline chemotherapeutic agent for many solid tumors including those of the breast however, dosage has to be carefully monitored to avoid the potentially life threatening complications associated with cardiotoxicity. Furthermore, in some cases tumors can acquire resistance to DOX greatly reducing its efficacy. In some cases these two factors can severely limit the clinical usage of this class of drugs. The mechanism of cardiotoxicity is unclear but it has been suggested that multiple processes are involved [1]. Mitochondrial failure has been suggested as a probable causative factor because DOX interacts with mitochondrial enzymes to induce highly reactive oxygen species (ROS) that immediately target nearby mitochondrial structural components including DNA to cause single and double strand breaks (SSBs and DSBs) [2], [3]. Moreover, DOX-induced ROS can also inactivate other biomolecules critical to mitochondrial function including lipids and proteins. Furthermore, as a chromosomal DNA damaging agent, DOX has been proposed to induce chromosomal DSB DNA damage by mechanisms other than ROS production including: 1) direct inhibition of type II topoisomerases [4]–[6]; 2) alkylation or intercalation with DNA [7]; 3) DNA crosslinking which inhibits unwinding and replication [8]; 4) or transcription inhibition [7]. Thus, DOX appears to be able to induce DSB damage by multiple mechanisms that could occur throughout the cell cycle including G1 and S phases.

The ability of tumors to simultaneously develop resistance to many drugs has been termed multidrug resistance (MDR) and frequently occurs following DOX treatment. Potential mechanisms for this acquired resistance include upregulation of transporters that promote drug efflux [9]–[11] as well as defects in downstream effector pathways including p53 [12] or Bcl-2 mediated apoptosis [13]. Altered expression of critical components within repair related pathways have also been found to confer resistance to DOX-induced DNA damage including type II topoisomerases [4], [5], [14], p53 [12], [15], DNA ligase IV and DNA-PK [16], 14-3-3sigma [17] and Rad51 [18]. Other components in pathways with no known repair function have also been implicated including ALDH4 [19], cathepsin D [20], Nrf2 [21]. Which of these genes or pathways, if any, are the most relevant for specific types of cancer remains uncertain.

To identify highly conserved targets that mediate resistance to DOX, many studies have successfully utilized the genetic accessibility of the model organism *Saccharomyces cerevisiae* [22]–[24], [5], [25]–[30], [31]–[38]. These studies have clearly implicated both the type II topoisomerases and the mitochondria as targets that mediate hypersensitivity to this cytotoxic drug. One study of particular interest was a genome-wide screen in the haploid deletion collection which identified 71 gene deletions that had enhanced sensitivity to DOX [38].

To further elucidate the mechanism of DNA damage resistance in *S. cerevisiae*, we screened the diploid deletion collection for mutants that are sensitive to doxorubicin. In this genome-wide screen, we identified 376 deletion mutants that are sensitive to the lethal and/or growth inhibitory effects of DOX compared to the wild type parental strain. This mutant collection is significantly enriched for deletions that show cross sensitivity to IR and/or G1 cell cycle defects. Our screen in the diploid organism identified 5-fold more DOX resistance genes (376 *versus* 71) than a similar genome-wide screen for doxorubicin sensitive mutants performed in the isogenic haploid strain [38]. Unlike haploids, diploids have the unique capability for recombinational repair of DSB damage prior to the completion of DNA replication and suggests we have identified genes that specifically affect repair of DOX-induced damage in G1 or early S phases.

To test this, we directly compared the relative sensitivity of diploid *versus* haploid deletion for genes that were identified in the diploid screen but not in the haploid screen. Concurrently, we screened for cross sensitivity to the S phase specific DNA damaging agents HU and MMS. All diploid strains examined demonstrated sensitivity to DOX and the S phase specific inhibitors HU or MMS. Of 30 mutants tested, 24 demonstrated enhanced sensitivity to doxorubicin as both a diploid and an isogenic haploid when compared to the repair competent WT strains. Thus the higher DOX doses used in the diploid screen was more effective in identifying DOX resistance genes. However, deletions of *BEM1, CTF4, CTK1, HFI1, NUP133* and *THO2* showed greatly enhanced sensitivity to DOX as a diploid when compared to the isogenic haploid stains. Subsequent characterization revealed that these gene deletions appear to affect G1 repair processes with (*CTF4, NUP133*, *CTK1* and *HFI1*) or without (*THO2* or *BEM1*) instability of the *MAT* locus. Thus the use of the diploid deletion collection has facilitated the detection of an extensive network of G1/S phase specific repair genes that confer overlapping resistance to DOX as well as IR and other agents. Many of these are highly conserved (76%) and form a large interactive network that associates with genes that impact on numerous cellular processes including mitochondrial function. Genetic defects and/or polymorphisms in these conserved DOX resistance genes may mediate cardiotoxicity in patients undergoing DOX chemotherapy or serve as biomarkers for therapeutic response to DOX chemotherapy in human tumors.

## Materials and Methods

### Yeast strains

Deletions of individual non-essential radiation resistance genes (or ORFs) were made in *MATa* (BY4741) and *MATα* (BY4742) haploid *S. cerevisiae* strains as part of The Saccharomyces Gene Deletion Project and subsequently mated to produce the isogenic diploid deletion strains. The diploid deletion strains were purchased in 96 well microtiter dishes from Open Biosystems and stored at −70°C. Isogenic *MATα* haploid deletion strains were obtained from the Yeast Model Systems Genomics Group at Duke University. *MAT*a haploid deletion strains used in this study were purchased from Open Biosystems.

### Doxorubicin and zymocin chemical genomic screening

A doxorubicin (DOX) stock solution (10 mg/ml in sterile H_2_0) was used to prepare DOX YPD agar plates at two concentrations (25 or 50 µg/ml). DOX was added to cooled YPD agar at the time of pouring and plates were allowed to solidify at room temperature and used immediately for screening the diploid deletion collection. Strains from the frozen deletion collection individually arrayed in 96 well dishes were thawed and aliquots (∼2 µl) were transferred using a multi (48) pin “pronging” device to YPD and YPD DOX. Concomitant with the DOX screen, zymocin screening of the deletion collection was also performed by replica pronging directly from the thawed 96 well dishes onto YPD plates containing 0, 33% or 66% crude zymocin. Zymocin containing YPD plates were made as previously described [39] (see below for brief description). DOX sensitive strains were identified after 2 days incubation at 30°C. Zymocin resistant deletions were identified following 1–2 days of incubation at 30°C. Zymocin sensitive strains were identified following 3 days incubation at 30°C.

Selected strains identified as DOX or zymocin sensitive or zymocin resistant in the primary screen were subsequently confirmed by growing individual isolates (and WT) in 200 µl of YPD in 96 well dishes for two days. These cells were serially diluted (5-fold) in liquid YPD and ∼2 µl of each dilution was replica plated by pronging to either YPD, YPD containing DOX or YPD containing zymocin. Resistance to zymocin was scored following 2 days incubation. Zymocin sensitivity was scored following 3 days incubation at 30°C.

Strains were screened for hydroxyurea (HU) and methyl methane sulfonate (MMS) sensitivity using a similar dilution plating procedure as previously described [40]. Briefly at least two individual isolates of each strain were grown in liquid YPD (200 µl) for two days at 30°C in 96 well microtiter dishes. Serial 5 fold dilutions of these stationary cell cultures were made in fresh YPD and ∼2 µl of each dilution was transferred to a control YPD plate and a YPD plate containing the chemical DNA damaging agent using the replica plating device described above. Inhibition of cell growth was determined after 24 and 48 h growth at 30°C.

### Zymocin preparation

Deletion strains were exposed to zymocin on plates either directly from the diploid deletion collection arrayed in 96 well dishes or using the dilution pronging technique described above. Alternatively, selected deletion strains and WT were grown for two days in liquid YPD (filter sterilized) in 96 well plates and serial 5 fold dilutions were made in water. Cells (∼2 µl of each dilution) were replica transferred to YPD and YPD+zymocin plates. YPD plates containing zymocin were made by growing *K. lactis* strain AWJ137 on filter sterilized liquid YPD for two days at room temperature. Briefly, two parts of a sterile YPD filtrate of conditioned medium from the 48 hr culture of the *K. lactis* strain was mixed with one part YPD agar to produce 66% zymocin plates. The 33% zymocin plates were made in a similar manner with the exception that 1 part conditioned medium was mixed with 2 parts liquid YPD agar which had been cooled following sterilization by autoclave. The final agar concentration was 2%. Plates were immediately poured and allowed to solidify at RT.

### Cell cycle progression analysis

WT and selected DOX sensitive deletion strains were examined for cell cycle progression following exposure to DOX as previously described [40], [41], [39]. Briefly, single unbudded (G1) cells from logarithmically growing cultures in YPD liquid cultures were arrayed into a 4×5 (20) cell grid pattern onto YPD and YPD plates containing DOX (50 µg/ml) using a Singer MSM dissecting microscope. Each grid was positioned such that all cells were visible within one field of view at 300× magnification. Cell cycle progression was determined by microscopic observations at hourly intervals and photographed using an Olympus Q-color 3 camera.

### Recombination assay

The PCR mediated gene conversion assay utilizing the *his3Δ1* allele as a target for recombination has been previously described [40]. Briefly, 1 µg of a PCR fragment that spans the internal deletion within the *his3Δ1* allele was transformed into WT and various diploid deletion strains [42] and the frequency of gene conversion of the *his3Δ1* allele to *HIS3* determined by plating to synthetic complete (SC) medium lacking histidine. To control for relative transformation efficiency, 200 ng of the plasmid pRS315 containing the *LEU2* selectable marker was co-transformed along with the PCR product containing the *HIS3* fragment before plating an aliquot of the transformation mix to SC medium lacking leucine. Relative gene conversion frequencies were normalized to that seen in WT.

### Mating-type determinations

WT and various diploid deletion strains were patched from single colony isolates or single colonies themselves grown on YPD plates were mated on fresh YPD plates to mating type tester strains 147 (*MATa pet8 met2 arg1 his7 met14 [KIL-k]*) and 148 (*MATα pet8 met2 arg1 his7 [KIL-k] disomic for XI: met14/MET14*) for 24 hours at 30°C. Mated isolates were subsequently replica plated to minimal and YPD media and allowed to grow for 24–48 hours at 30°C.

## Results

### Checkpoint, recombinational repair and mitochondrial functions are required for doxorubicin resistance in diploid yeast strains

**Figure 1:**
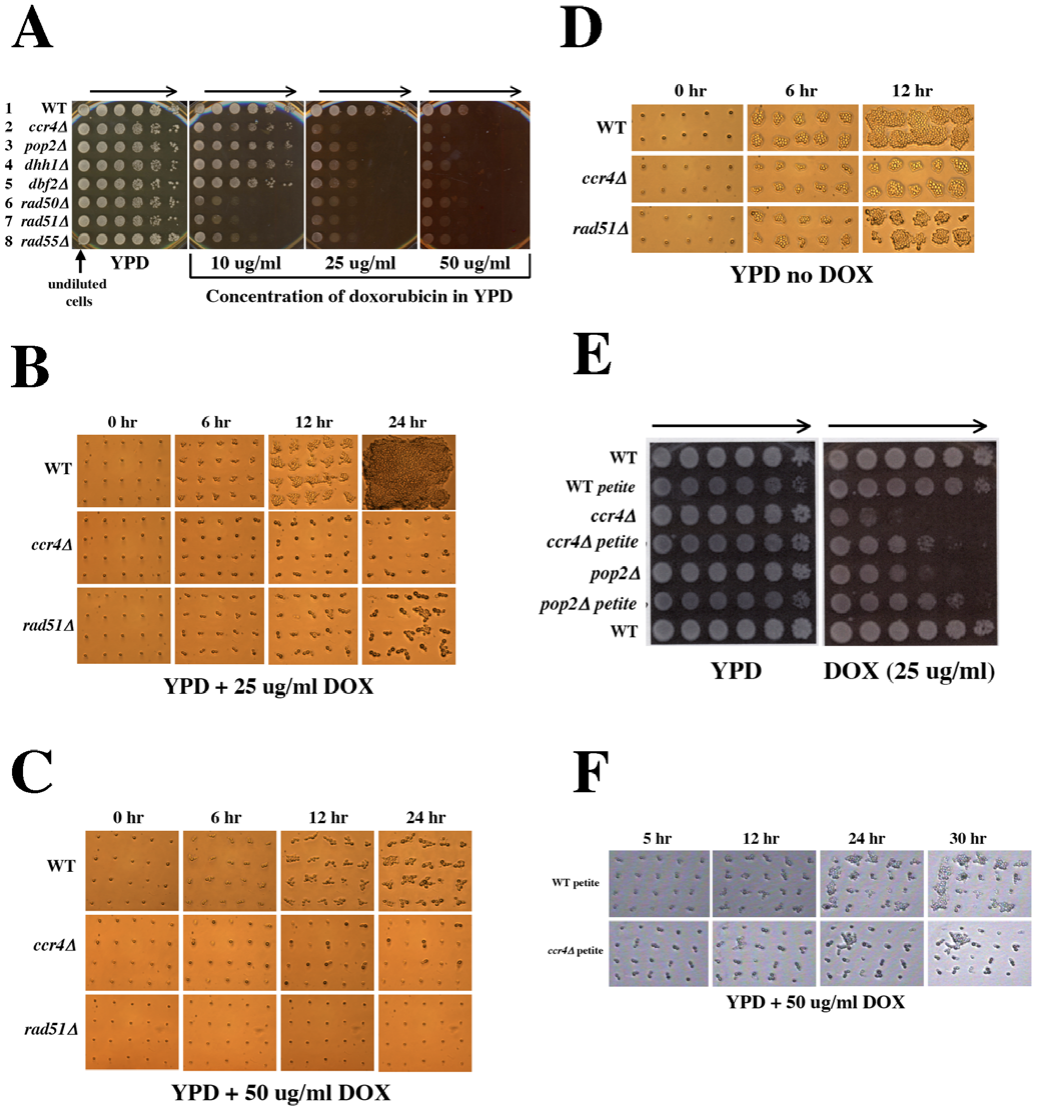
Checkpoint, recombinational repair and mitochondrial functions are required for doxorubicin resistance in diploid yeast strains. (A) Isogenic ionizing radiation (IR) sensitive diploid deletion strains were grown at 30°C for two days in liquid YPD medium in 96 well plates. Serial 5-fold dilutions were made in sterile water and 2 µl aliquots were replica plated to YPD solid medium with and without the indicated doses of doxorubicin. Plates were subsequently incubated for 3 days at 30°C. Arrows indicate the direction of decreasing cell concentration. When compared to WT (row 1), defects in genes within the CCR4-NOT complex (rows 2–5) confer checkpoint adaptation functions and show intermediate sensitivity to doxorubicin. Defects in members of the RAD52 recombination repair group (rows 6–8) are required for double strand break repair and are hypersensitive to doxorubicin. (B) Diploid WT, *ccr4Δ* and *rad51Δ* cells were grown to logarithmic phase in liquid YPD and individual unbudded (G1) cells were plated in a 5×4 cell grid pattern to YPD containing doxorubicin at the indicated dose within one microscopic field of view using a Singer MSM micromanipulator. DOX-induced inhibition of cell cycle progression in G1 and G1/S phases of the cell cycle was monitored by photomicroscopy at hourly intervals. Cells were incubated at 30°C during cell cycle progression analysis. (C) Similar to panel B except individual unbudded cells were gridded onto YPD plates containing 50 µg/ml DOX. (D) Similar to panel B except individual unbudded cells were gridded onto YPD medium without doxorubicin to demonstrate normal cell cycle progression and 100% cell viability. (E) Isogenic respiratory competent and petite WT, *ccr4Δ* and *pop2Δ* strains were grown in liquid YPD and serially diluted in 96 well plates as described in panel A. Both the *ccr4Δ* and *pop2Δ* petite strains show enhanced resistance to the lethal effects of doxorubicin. (F) Similar to panel C except individual unbudded cells were from petite strains that lacked respiratory function.

We have described a large interactive network of ionizing radiation resistance genes in which the CCR4-NOT complex plays a key role [39]. Deletions within the CCR4 damage response network are sensitive to IR-induced DSB damage as diploids but not as haploids and appear to function as checkpoint adaptation genes. Moreover, lethality induced by doxorubicin (DOX) is mediated indirectly by reactive oxygen species (ROS) generated within the mitochondrion or through inhibition of topoisomerase II both of which induce DSB damage. Thus, similar to IR, DOX-induced DSB damage requires the recombinational repair activity of the RAD52 group of recombinational repair genes. In order to establish an appropriate screening concentration for exposure of the diploid deletion strain collection to DOX, we examined selected IR sensitive mutant strains within the CCR4-NOT complex (*ccr4Δ, dhh1Δ, pop2Δ* and *dbf2Δ)* which have moderate IR sensitivity and recombination repair deficient strains that are extremely IR sensitive (genes within the RAD52 epistasis group) to increasing concentrations of DOX using a multi-pin replica plating device (10, 25 and 50 µg/ml; Fig. 1A). A dose dependent decrease in survival was observed for all of the mutant strains with the recombination deficient strains demonstrating the greatest sensitivity to DOX. For mutant strains that have moderate sensitivity to agents that induce DSB damage (such as those within the CCR4 damage response pathway), a dose of 50 µg/ml was required to see a decrease in survival for undiluted cells, (*i.e.,* similar to the conditions expected for screening the deletion collection directly from the arrayed 96 well plate format). For strains hypersensitive to DOX (such as those mutants within the RAD52 group of repair genes), a dose of 25 µg/ml was adequate for observing decreased survival of undiluted cells. However, at a dose of 10 µg/ml the undiluted DSB sensitive deletion strains did not show a significant decrease in survival when compared to WT. Therefore, in order to identify gene deletions that confer both moderate and severe hypersensitivity to the lethal effects of DOX, we screened the arrayed diploid deletion collection at both 50 and 25 µg/ml of DOX in YPD medium.

Deletion of genes within the CCR4 damage response network results in cell cycle checkpoint adaptation defects during the G1 to S phase transition following DSBs or replication stress [39]. In order to initially characterize the cell cycle response to DOX, we examined cell cycle progression of unbudded (G1) checkpoint deficient diploid *ccr4Δ* to DOX at 25 µg/ml (Fig. 1B) and 50 µg/ml (Fig. 1C). These were compared to the repair proficient WT and recombination deficient *rad51Δ* diploid cells exposed to DOX at the same doses. Mutant *ccr4Δ* cells clearly demonstrated a severe cell cycle progression defect when compared to WT at both doses (Fig. 1B, C). Strikingly when exposed in G1 to either low (25 µg/ml) or high concentration of DOX (50 µg/ml), many *ccr4Δ* cells (40 and 70% respectively) failed to progress into S phase and arrested permanently as single cells in G1. Following prolonged exposure to DOX, most of these diploid *ccr4Δ* cells underwent lysis in a manner similar to that seen following exposure to hydroxyurea [39]. This DOX-induced cellular lysis was observed in both G1 arrested cells and those that did progress into S phase (Fig. 1B, C). A fraction of *ccr4Δ* cells (20%) failed to arrest at either G1 or in S phase and formed microcolonies of 3 or more cells following DOX exposure at the low dose (Fig. 1B). Similar to that seen following IR exposure, recombination deficient *rad51Δ* cells transited from G1 to S phase and arrested as large budded cells following exposure to low doses of DOX (Fig. 1B). The majority of these recombination deficient cells (80%) adapted to the DOX-induced cell cycle arrest and resumed cycling to form microcolonies of 3 or more cells. At the higher DOX dose the recombination deficient *rad51Δ* cells failed to progress and arrested permanently in G1 (Fig. 1C). WT diploid cells progressed rapidly into microcolonies following exposure to high doses of DOX (Fig. 1C). In the absence of DOX, all strains showed high viability and rapid cell cycle progression when plated to YPD as single G1 cells (Fig. 1D). Thus the checkpoint functions associated with CCR4-mediated damage responses are required for resistance to DOX which, in the absence of Ccr4 induced a prolonged G1 arrest followed by cellular lysis. A few *ccr4Δ* cells escaped G1 arrest and proceeded into S phase where a permanent cell cycle arrest and lysis was observed. A dose dependent arrest phenotype was observed for the *rad51Δ* strain in which a permanent G1 arrest occurred at the high DOX dose. At low DOX doses, *rad51Δ* cells were capable of adapting to the DOX-induced damage and resumed cell cycling to form microcolonies. The hypersensitivity of diploid deletion mutants within members of the RAD52 epistasis group of repair genes clearly implicates DOX-induced DSBs as a major contributor to lethality that require repair by mechanisms of homologous recombination.

### Doxorubicin-induced ROS mediates lethality in G1

Interaction of DOX within mitochondria has been proposed to result in the generation of reactive oxygen species (ROS) that contributes to cellular lethality. To examine whether mitochondrial processing of DOX contributes significantly to lethality and/or checkpoint arrest in diploid yeast we “cured” the WT, *ccr4Δ* and *pop2Δ* diploid strains of functional mitochondria by generating petite mutants that fail to grow using glycerol as a carbon source. The relative survival following DOX exposure of the isogenic WT, *ccr4Δ* and *pop2Δ* strains with and without mitochondrial function was determined (Fig. 1E). Although the loss of mitochondrial functions enhanced DOX resistance in both the *ccr4Δ* and *pop2Δ* mutants it did not totally eliminate DOX-induced lethality suggesting that survival following DOX exposure is only in part dependent on the maintenance of functional mitochondria. Moreover, this suggests that multiple mechanisms are responsible for DOX-induced lethality in diploid yeast.

In order to determine if the loss of mitochondrial functions affected cell cycle responses to DOX we examined cell cycle progression of logarithmic diploid WT, *ccr4Δ* (Fig. 1F) and *pop2Δ* (data not shown) G1 cells without functional mitochondria (*i.e*., petites) following exposure to DOX. In the absence of functional mitochondria, most *ccr4Δ* and *pop2Δ* cells rapidly progressed from unbudded (G1) cells into budded (S phase) cells (Fig. 1F). In petite diploid *ccr4Δ* cells that were respiratory deficient, only 15% (3/20) of G1 cells exposed to DOX remained permanently arrested in G1 as opposed to those in isogenic respiratory proficient *ccr4Δ* cells in which 75% (15/20) remained permanently arrested in G1 (Fig. 1C). Similarly, a prolonged DOX-induced G1 arrest was more evident in diploid *pop2Δ* strains that were respiratory proficient (50% arrested in G1) as compared to those that were respiratory deficient (20% arrest in G1; data not shown). Thus the prolonged DOX-induced G1 arrest observed in strains defective in components of the CCR4 damage response required the presence of functional mitochondria and implicates ROS as a DNA damage intermediate that elicits damage in G1.

### A genome-wide diploid screen identifies 376 gene deletions that are sensitive to doxorubicin

The diploid deletion collection has been useful for identifying ionizing radiation (IR) repair associated genes that function specifically in G1 [39]. To identify new DOX resistance genes, we utilized a “spot” testing procedure identical to that which we had previously used to identify IR resistance genes. In order to see significant lethality for undiluted cells, the diploid deletion collection was simultaneously screened at two concentrations (Fig. 1A; 25 and 50 µg/ml). Deletion strains arrayed in the 96 well format were replica pronged to YPD plates containing the two concentrations of DOX and growth inhibition scored by comparison to growth on YPD plates without DOX. Strains that exhibited the most hypersensitivity to DOX showed complete inhibition and no residual growth on both the 25 and 50 µg/ml DOX were scored as a “3”. Deletions that showed moderate DOX hypersensitivity demonstrated complete inhibition at 50 µg/ml DOX but only partial growth inhibition on 25 µg/ml and were scored as a “2”. Strains that were slightly hypersensitive to DOX were those showing complete growth inhibition on 50 µg/ml plates but little or no growth inhibition on 25 µg/ml DOX. These were scored as a “1” (Supplementary Table S1). In addition to this scoring scheme other deletion strains lacked complete killing, but instead showed a slowed growth response to DOX on YPD plates when compared to that observed for the majority of deletion strains that were resistant to DOX and displayed rapid growth during initial DOX screening on YPD (Supplementary Table S2).

A total of 376 diploid deletion strains demonstrated either hypersensitivity (n = 209) or reduced growth rate (n = 167) when exposed to DOX. This represents ∼8% of the non-essential genes represented within the diploid deletion collection. Remarkably, this collection of DOX sensitive gene deletions is significantly larger (>5 fold) than that found in a similar screen using the isogenic haploid deletion collection from which only 71 deletion strains were identified (Table 1; Supplementary Tables S1 and S2). Of the 71 mutants identified in the haploid screen, we identified 43 (61%) deletions as being sensitive to DOX in our diploid screen. Of the remaining 28 haploid mutants not found in the diploid screen, we re-examined 10 of the most DOX sensitive haploid deletions detected (those described as being SSS or SS [38]). Using dilution plating to 50 and 25 µg/ml DOX, we found that diploid *vma21Δ, hom6Δ, trp1Δ* and *mac1Δ* (*i.e.* 40% of strains tested) were sensitive to DOX as a diploid (data not shown) and therefore missed using our screening protocol in the diploid deletion collection. The other six diploid deletion strains (*afg3Δ, erg3Δ, mrpl6Δ, mrpl37Δ, vps36Δ* and *yor199wΔ*) were resistant to DOX as a diploid suggesting these may represent a subset of haploid-specific DOX-resistance genes. These six gene deletion strains were not examined further.

**Table 1:** A genome-wide screen in the yeast diploid deletion collection identifies 209 doxorubicin resistance genes enriched for those that show cross sensitivity to zymocin, ionizing radiation, loss of G1 size control and oxidative damage.

Zymo^1^	IR^2^	G1 size^3^	Oxid^4^	Yeast DOX resistance gene	Conserved human ortholog
S	S	S	S	ASF1, CCR4, DBF2, HFI1, MMS22, POP2, RAD50, RTT109, YDJ1	ASF1A, CNOT6, STK38L, none, ANKRD12, CNOT8, RAD50, RTT109, HSP40
			R	ADK1, AKR1, ARP5, BEM1, MDM20, RPB9	AK2, ZDHHC17, ACTR5, SH3PXD2B, C12orf30, POLR2I
		R	S	TOP3, TPS1, YAF9, YEL033W	TOP3A, none, YEATS4, none
			R	CLC1, CTF4, DHH1, DOC1, GRR1, GUP1, NOT5, OCH1, RAD51, RAD52, RAD54, TSR2, TUP1, VMA7, XRS2, YLR235C	CLTA, WDHD1, DDX6, ANAPC10, FBXL20, HHATL, CNOT3, none, RAD51, RAD52, RAD54L, TSR2, WDR5, ATP6V1F, NBS1, none
	R	S	S	GAL11, IFM1, IMP2', MSE1, MSM1, PEP3, PHO85, RNR4, ROX3, RTS1, SNF5, SUV3, SWI6	MLL2, MTIF2, SFRS12, EARS2, MARS2, VPS18, CDK2, RRM2, none, PPP2R5D, MLL2, SUPV3L1, AKAP9
			R	ADH1, ANP1, BEM4, BUD25, IES6, MIP1, MNN9, MNN10, MSD1, PIN4, RNR1, SHP1, SPT7, TCO89, VPS34	ADH1B, TNRC6A, none, none, C18orf37, POLG, none, none, DARS2, MLL5, RRM1, NSFL1C, BAZ1A, DSPP, PIK3C3
		R	S	BUD23, ERG4, LST4, PFK26, PGD1, PHO2, PKR1, PTC1, REG1, SNF2, SNF6, SOD1, SWI3, TAT1, VAN1, VMA2, VMA4, YJL175W	WBSCR22, LBR, LOC100133790, PFKFB3, MUC7, PITX1, none, PPM1B, DSPP, SMARCA2, none, SOD1, SMARCC2, SLC7A14, none, ATP6V1B2, ATP6V1E1, none
			R	ACO1, BUD16, CCW12, CUP5, DOA4, ERG6, GAS1, HEX3, HOM2, HOM3, HTZ1, KHA1, MSY1, PER1, RRN10, SAC7, SER2, SLM4, NAB6, VPS64, VMA5, YOL050C, YOR331C, YPL205C	ACO2, PDXK, LOC100132635, ATP6VOC, USP8, TGS1, MUC21, HRNR, none, none, H2AFV, TMCO3, YARS2, PERLD1, none, ARHGAP6, PSPH, none, none, SLMAP, ATP6V1C1, none, none, none
R	S	S	S	PAT1, SLX8, YJL188C	PATL1, RNF10, none,
			R	BCK1, FUN12, HPR1, LGE1, NPL3, PLC1, THO2	MAP3K3, EIF5B, THOC2, FLG, HNRNPR, PLCD4, THOC2
		R	S	RSA1	AKAP9
			R	ADE12, GON7, LSM7, MMS4, NUP133, RAD55, RAD57, RAD59, VPH2, YDL041W, YDR433W, YKL118W, YML009C-A	ADSSL1, none, LSM7, none, none, RAD51L3, RAD51L1, RAD52, none, none, none, none, none
	R	S	S	DBP7, ECM33, MSN5, RPL35A, RPL43A, SAC1, SAC3, SIN3, SSZ1, UAF30	DDX31, MUC21, XPO5, RPL35, RPL37A, SAC1L, MCM3AP, SIN3A, HSPA8, SMARCD1,
			R	ASC1, BUD22, CTK3, FYV5, HIT1, KRE6, MET7, OPI11, PRO1, RPL39, RPS10A,	GNB2L1, LOC100133599, none, none, none, DSPP, FPGS, none, ALDH18A1, LOC100133222, RPS10,
		R	S	CBC2, GCR2, HAL5, KCS1, LSM1, NSR1, PDR1, RPL27A, RPS4A, RPS11B, SAT4, SIN4, VMA13, YAR1	NCBP2, MUC21, PRKAA1, IHPK3, LSM1, NCL, none, RPL27, RPS4X, RPS11, CHEK1, none, ATP6V1H, FEM1C
			R	AKL1, CKB1, CKB2, CTI6, YPL182C, CTK1, EDC3, EGD1, ERV41, GET1, HEM14, HHF1, MDM35, MMS1, MTQ2, NEW1, NFI1, PSK2, PUS1, PUS7, RDS2, RIS1, RPA49, RPL12A, RPL13B, RPL20B, RPP1A, RTG1, SER1, SPT20, TAF14, TCM62, TFP3, THP1, TRK1, VMA6, YCL007C, YDR049W, YGR160W, YNL140C, YOL046C, YOR152C, YPL260W, YPL261C	AAK1, CSNK2B, CSNK2B, CYLC1, POU2F1, CRKRS, ATP6V1D, BTF3L4, ERGIC2, none, PPOX, HIST1H4A, TRIAP1, none, N6AMT1, GCN20, PIAS4, PASK, PUS1, PUS7, FAM135A, HLTF, POLR1E, RPL12, RPL13, RPL18A, RPLP1, none, PSAT1, none, MLLT3, HSPD1, ATP6VOD1, PCID2, DSPP, ATP6VOD1, none, ANKZF1, LOC645490, none, none, ANKRD26, none, none

1 Resistance to the G1 specific toxin zymocin was determined in a screen that was performed in parallel to that for the identification of DOX resistance mutants. A total of 806 diploid deletion strains (16.6% of nonessential genes) were found to be hypersensitive to zymocin. A total of 106 DOX^S^ deletion mutants (50.7%) were found to be cross sensitive to the lethal effects of zymocin. This is 3 fold greater than that expected by chance alone.

2 A total of 204 ionizing radiation resistance genes (4% of nonessential genes) were identified in the diploid deletion collection as previously described [40], [39]. A total of 59 DOX^S^ deletions (28.6%) were found to overlap with those that were identified as IR resistance genes. This is 7 fold greater than that expected by chance alone and suggests that DSBs are a significant component of the spectrum of lesions induced by DOX in *S. cerevisiae.*

3 Approximately 500 gene deletions (∼10% of nonessential genes) in the haploid deletion collection were found to significantly affect cell size control that is determined in G1 and regulated by the checkpoint at “START” [43], [44]. A total of 74 DOX sensitive mutants (35.4%) were found to overlap with those that affect cell size control. This is 3.5 fold greater than that predicted by chance alone.

4 A total of 456 deletion mutants in the haploid deletion collection (9.4% of nonessential genes) were identified that demonstrated enhanced sensitivity to oxidative DNA damaging agents [60]. A total of 71 DOX^S^ mutants (31%) were found to overlap with those determined to be sensitive to oxidative damage. This is 3.3 fold greater than that predicted by chance alone and suggests that oxidative damage lesions are a significant component of the spectrum of lesions.

### Doxorubicin sensitive mutants are enriched for genes required for G1-dependent functions

DOX is a well-characterized chemotherapeutic that induces DNA damage by multiple mechanisms including the production of ROS by interaction with the mitochondria, direct inhibition of topoisomerase II or direct DNA interactions (by intercalation, alkylation and/or crosslinking). All of these processes are known to induce DSB damage. Therefore, it is not surprising that a subset of DOX sensitive genes significantly overlap with those that show sensitivity to IR and oxidative damage induced by H_2_O_2_ and other chemicals that act in G1 (Table 1; Supplementary Tables S1 and S2). Of the 376 DOX sensitive diploid mutants identified, 24.5% (92 deletions) had been previously found in our genome-wide screen for IR resistance genes in the diploid background including those required for the recombinational repair of IR-induced DSBs (*RAD50, RAD51, RAD52, RAD54, RAD55, RAD57* and *RAD59*). This overlap is 6 fold more than would be expected by chance alone. Furthermore, 33% of the DOX sensitive mutants (124 deletion mutants) overlap with those that confer resistance to oxidative stress damage including sod*1Δ*. This is >3 fold more than would be expected by chance alone and confirms that a significant amount of the lethality induced by DOX in yeast can be attributed to lesions indirectly induced by ROS. Moreover, highly conserved mitochondrial associated gene deletions were also overrepresented among the DOX-sensitive mutants (Supplementary Tables S1 and S2) suggesting that defects in mitochondria associated functions contribute to DOX induced ROS mediated lethality.

Among the diploid DOX resistance mutants, many (24.3%) overlap with those that were found to affect cell size control (Table 1; Supplementary Tables S1 and S2), a function that is regulated in the G1 phase of the cell cycle at START [43], [44]. This enrichment was >2.6 fold greater than that expected by chance alone and suggests that many DOX genes may function within G1 to confer DNA damage resistance. Finally, the subset of DOX sensitive deletion mutants were greatly enriched for mutants that showed G1 cell cycle defects either spontaneously or following exposure to the oxidizing agent linoleic acid hydroperoxide (LoaOOH) that arrests cells in G1 [45]. In fact 55% (26/47) of the deletion mutants that failed to arrest in G1 following LoaOOH exposure were sensitive to DOX suggesting that defects in G1 associated checkpoint control may represent a significant proportion of the DOX resistance genes identified in the diploid yeast screen. This also indicates that a substantial number of lethal DOX-induced lesions are inflicted during the G1 phase in diploid yeast.

Zymocin is a toxin secreted by the yeast *K. lactis* and has been shown to induce a prolonged lethal G1 arrest in *S. cerevisiae* [46], [47]. We previously determined that diploid IR sensitive deletions were enriched for those that were also sensitive to the toxin zymocin [39] and defects in the G1 associated DNA damage checkpoint mutant *HRR25* confer zymocin resistance [47]. We therefore concomitantly screened for sensitivity to zymocin during the screen for DOX. In order to initially determine the relative sensitivity of diploid deletion mutants to the toxic action of zymocin, we simultaneously exposed freshly thawed cells plated directly from the deletion collection to two different concentrations of zymocin as previously described [39]. Strains that exhibited the greatest hypersensitivity to zymocin-induced growth inhibition upon initial plating showed complete inhibition on both 33% and 66% zymocin and were scored as a “3”. Deletions that showed moderate zymocin sensitivity demonstrated complete inhibition on 66% zymocin but only partial or no growth inhibition on 33% plates and were scored as a “2”. Strains with slight zymocin sensitivity were classified as those showing partial growth inhibition on 66% plates with little or no inhibition on the 33% plates. These were scored as a “1”. We identified a total of 806 gene deletions that demonstrated enhanced sensitivity to zymocin (Westmoreland et al., manuscript in preparation). Of these, 202 (25%) were scored as hypersensitive, 396 (49%) were moderately sensitive and 208 (26%) were slightly sensitive to zymocin.

Among the zymocin sensitive diploid deletion strains identified in the primary screen, 103 were found to overlap with our previously described set of IR sensitive diploid deletion strains [40], [39]. A further 12 ionizing radiation sensitive deletion strains were found to confer zymocin resistance. Therefore, from the primary zymocin screening 58% (115/200) of the previously identified IR resistance genes were found to confer altered sensitivity to zymocin when deleted. Moreover, 45% of the DOX sensitive deletion strains (169/376) were found to be sensitive to zymocin-induced lethality (Table 1; Supplementary Tables S1 and S2). This suggests that the genetic pathways responsible for zymocin, IR and doxorubicin resistance significantly overlap. Furthermore, since the primary lesion responsible for DOX and IR-induced lethality is unrepaired DSB damage, this implies that zymocin-induced cytotoxicity may also result from the induction of persistent unrepaired DSB. Since zymocin is known to function in G1, the overlap between the zymocin responsive gene network and those genes that mediate IR and DOX resistance suggests that a significant fraction of DOX resistance similarly occurs in G1.

### Identification of diploid-specific doxorubicin resistance genes

**Figure 2:**
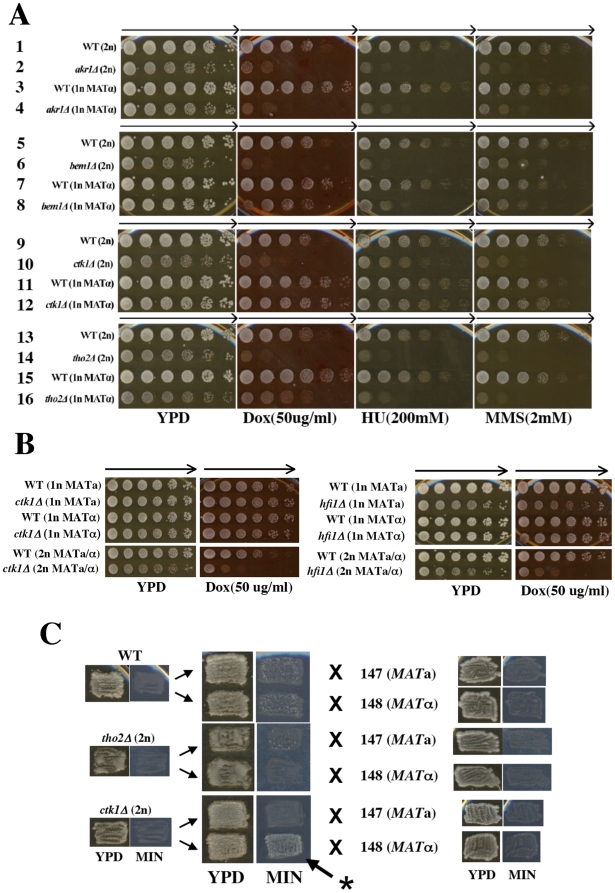
Identification of diploid-specific doxorubicin resistance genes. (A) Haploid (1n *MATα*) yeast deletion strains (rows 3, 4, 7, 8, 11, 12, 15, 16) were compared to their isogenic diploid (2n) deletion counterparts (rows 1, 2, 5, 6, 9, 10, 13, 14) for enhanced hypersensitivity to doxorubicin (DOX), hydroxyurea (HU) and methyl methanesulfonate (MMS) relative to wild type (WT; rows 1, 3, 5, 7, 9, 11, 13, 15) at the indicated concentrations in YPD agar medium. Cells were grown, diluted and plated as described in Fig. 1A. Arrows indicate direction of decreasing cell concentration. Deletion of *BEM1*(rows 6, 8), *CTK1* (rows 10, 12) or *THO2* (rows 14, 16) show enhanced sensitivity to the lethal effects of doxorubicin as a diploid. Deletion of *AKR1* demonstrated hypersensitivity to doxorubicin in both the diploid (row 2) and haploid (row 4) derivatives. All deletion strains with the exception of the haploid *ctk1Δ* (above) and *hfiΔ* (not shown) strains demonstrated hypersensitivity to HU and MMS when compared to WT. (B) Diploid specific hypersensitivity of *ctk1Δ* and *hfi1Δ* strains to doxorubicin-induced cytotoxicity in the diploid BY4743 background as compared to the isogenic BY4741 (*MAT*a), BY4742 (*MAT*a) haploid backgrounds. Dilution plating conditions were similar to that as described in Fig. 1A. (C) Some diploid-specific deletion strains demonstrate enhanced mating capability as diploids. All diploid-specific gene deletions were examined for the ability to mate to the haploid mating type tester strains147 (*MATa*) or 148 (*MATα*). WT diploid strains are non-maters. Some (*ctk1Δ, hfi1Δ, nup133Δ* and *ctf4Δ*) but not all (*tho2Δ, bem1Δ, mft1Δ, thp1Δ* and *thp2Δ*) diploid strains showed enhanced capability for mating and subsequent growth on minimal (MIN) agar medium. Representative diploid deletion strains which show enhanced mating capability (*ctk1Δ*) or no enhanced mating capability (*tho2Δ*) by growth on MIN medium (arrow *) are depicted.

The overlapping sensitivity of our diploid DOX sensitive deletion strains to the G1 specific toxin zymocin as well as with mutants sensitive to oxidative damage and those that regulate cell size control in G1 suggests that a significant fraction of the lethal activity of DOX occurs in the G1 phase of the cell cycle. Furthermore, as compared to haploids, diploid yeast are capable of repairing DSB via recombination in G1 due to the availability of a chromosome homolog. This suggests that among the mutants identified exclusively in the diploid screen and absent in the haploid screen, some may exert repair activity specifically in G1. Alternatively, since we utilized a dose higher than that used in the haploid screen (50 and 25 µg/ml as compared to ∼11 µg/ml), this may have allowed the identification of more DOX resistance genes. To test this directly, we compared (relative to WT) the haploid and diploid DOX sensitivities for 26 mutants detected in the diploid DOX screen but not found in the haploid DOX screen as well as 4 diploid sensitive mutants (*adk1Δ, bem1Δ, hfi1Δ* and *rtt109Δ*) that were also detected in the haploid DOX screen (Fig. 2A; Table 2). In addition, we selected these mutants based on their known cross sensitivity to IR as diploids [40], [39]. Among these we found that *akr1Δ, arp5Δ, ccr4Δ, dbf2Δ, dhh1Δ, hpr1Δ, lge1Δ, lsm7Δ, mdm20Δ, mms4Δ, mms22Δ, nup133Δ, och1Δ, pat1Δ, plc1Δ, pop2Δ, rad54Δ, rad59Δ, rpb9Δ, slx8Δ, tho2Δ, tup1Δ, vma7Δ* and *yaf9Δ* (Fig. 2A, Table 2) all showed sensitivity to DOX in the isogenic haploid strain backgrounds when compared to WT. With the exception of *ctf4Δ, ctk1Δ* and *hfi1Δ*, all of these haploid *MATα* mutants showed cross sensitivity to the S phase specific DNA damaging agents HU or MMS indicating that these deletions have repair associated defects that extend into S phase. Interestingly, some deletions (*mms4Δ, tup1Δ* and *yaf9Δ*) were sensitive to MMS but completely insensitive to HU as both haploid and diploid genotypes (Table 2).

**Table 2:** Enhanced sensitivity of isogenic diploid and haploid deletion strains to doxorubicin, hydroxyurea (HU) or methyl methanesulfonate (MMS) when compared to repair competent (WT) parental strain.

Yeast deletion^1^	DOX^2^		HU^3^		MMS^4^	
	2n	1n	2n	1n	2n	1n
*adk1Δ* *	>SSS	>SSS	>SSS	>SSS	S	SS
*akr1Δ*	>SSS	>SSS	>SSS	>SSS	>SSS	SSS
*arp5Δ*	SS	>SSS	SS	S	>SSS	>SSS
***bem1Δ*** *	**>SSS**	**S**	**>SSS**	**SSS**	**SS**	**SS**
*ccr4Δ*	>SSS	>SSS	>SSS	>SSS	>SSS	SSS
***ctf4Δ***	**SSS**	**-**	**SS**	**-**	**>SSS**	**-**
***ctk1Δ***	**SSS**	**-**	**S**	**-**	**>SSS**	**-**
*dbf2Δ*	SSS	SSS	>SSS	>SSS	>SSS	>SSS
*dhh1Δ*	SSS	SSS	>SSS	>SSS	>SSS	>SSS
***hfi1Δ*** *	**>SSS**	**-**	**>SSS**	**-**	**SS**	**-**
*hpr1Δ*	SSS	>SSS	SSS	SSS	>SSS	>SSS
*lge1Δ*	SSS	SSS	S	S	S	SSS
*lsm7Δ*	SSS	SSS	>SSS	>SSS	SSS	SSS
*mdm20Δ*	SS	SSS	S	S	SSS	SSS
*mms4Δ*	SSS	SSS	-	-	>SSS	>SSS
*mms22Δ*	>SSS	>SSS	SSS	SSS	>SSS	>SSS
***nup133Δ***	**>SSS**	**S**	**S**	**S**	**SSS**	**SSS**
*och1Δ*	>SSS	>SSS	S	S	S	S
*pat1Δ*	SS	SS	S	S	SSS	SSS
*plc1Δ*	>SSS	>SSS	>SSS	>SSS	S	S
*pop2Δ*	>SSS	>SSS	SSS	>SSS	SS	SSS
*rad54Δ*	>SSS	>SSS	>SSS	>SSS	>SSS	>SSS
*rad59Δ*	>SSS	>SSS	S	S	>SSS	>SSS
*rpb9Δ*	>SSS	SS	>SSS	>SSS	SS	>SSS
*rtt109Δ* *	>SSS	>SSS	SS	SSS	SSS	SSS
*slx8Δ*	>SSS	>SSS	SSS	SSS	SS	-
***tho2Δ***	**>SSS**	**S**	**>SSS**	**>SSS**	**>SSS**	**>SSS**
*tup1Δ*	>SSS	>SSS	-	-	SSS	>SSS
*vma7Δ*	>SSS	>SSS	SS	SS	>SSS	>SSS
*yaf9Δ*	SS	SS	-	-	SSS	SSS
n = 30		27		24		26
**Other THO-associated genes**						
***mft1Δ***	**SS**	**-**	**S**	**S**	**S**	**S**
***thp1Δ***	**>SSS**	**SS**	**SSS**	**SSS**	**>SSS**	**>SSS**
***thp2Δ***	**SSS**	**S**	**S**	**S**	**SS**	**-**

* These deletion strains were detected in the haploid DOX screen [38].

1 Yeast deletions identified in the diploid deletion DOX screen were cross sensitive to ionizing radiation (see [40], [39]) and Table 1. Bold indicates deletion strains that showed diploid-specific enhanced sensitivity to DOX.

2 Relative sensitivity of the diploid (2n) versus haploid (1n) deletion strains to DOX was determined at a concentration of 50 ug/ml. Cells were grown in liquid YPD for two days and serial 5 fold dilutions made in sterile water. Two ul aliquots were then spotted to YPD and the DOX plates and allowed to grow for 3 days. >SSS denotes an enhanced sensitivity for a given deletion mutant that was greater than 125 fold over that observed for the isogenic WT of the same ploidy; SSS denotes a 125 fold enhanced sensitivity of the mutant when compared to WT; SS denotes a 25 fold enhanced sensitivity of the mutant when compared to WT; S denotes a five fold enhanced sensitivity of the mutant when compared to WT; “ - “ denotes no enhanced sensitivity of the mutant when compared to WT.

3 Relative sensitivity to hydroxyurea (HU) was determined at 200 uM.

4 Relative sensitivity to methyl methanesulfonate (MMS) was determined at 2 uM.

For six of the deletion strains examined (*bem1Δ, ctf4Δ, ctk1Δ*, *hfi1Δ, nup133Δ* and *tho2Δ*), the diploid deletions showed hypersensitivity to DOX when compared to WT (≥125-fold) whereas the isogenic haploid derivatives showed little (*bem1Δ, hfi1Δ, nup133Δ* and *tho2Δ)* or no (*ctf4Δ* and *ctk1Δ*) sensitivity compared to WT (Fig. 2A; Table 2). For three of these mutants that showed diploid-specific sensitivity to DOX (*ctf4Δ, ctk1Δ* and *hfi1Δ*), a similar diploid-specific hypersensitivity to cell killing was observed in response to the S phase specific DNA damaging agents HU and MMS. For the remaining three deletions that showed diploid-specific hypersensitivity (*bem1Δ, nup133Δ* and *tho2Δ)*, no diploid-specific hypersensitivity to HU or MMS was observed. Instead, both the haploid and diploid mutant derivatives had similar sensitivity to HU and MMS when compared to their WT counterparts. Interestingly, one diploid deletion (*slx8Δ*) showed enhanced sensitivity (25-fold) to the lethal effects of MMS when compared to the WT whereas the haploid deletion did not and both the diploid and haploid deletions showed similar hypersensitivity to DOX (>125 fold) and HU (125-fold; Table 2). Since, the haploid *MAT*a variant of the *hfi1Δ* strain was described as hypersensitive to DOX, and both the *ctk1Δ* and *hfi1Δ* diploids display enhanced levels of mating with *MAT*α tester strains (see below), we compared the relative sensitivity of the *MAT*a haploid derivatives to DNA damage when compared to the WT counterpart. In contrast to the hypersensitivity of the diploid deletions to DOX, both the *MAT*a and *MAT*α haploid variants of the *hfi1Δ* and *ctk1Δ* strains demonstrated little or no sensitivity to DOX (Fig. 2B) suggesting that under our experimental conditions resistance to DOX mediated by *CTK1* and *HFI1* is primarily a diploid specific event in the BY4743 strain background. Moreover, we remade the *ctk1Δ* deletion in the *MAT*a haploid BY4742 strain background and confirmed that it was not sensitive to DOX (data not shown) suggesting that the haploid *ctk1Δ* strains (Fig. 2B) had not acquired genetic suppressors of DOX-induced toxicity.

Both *HPR1* and *THO2* have been identified as genes which encode components of the THO complex that are required for transcription elongation and participate in mitotic recombination processes [48], [49]. Surprisingly, the *hpr1Δ* and *tho2Δ* mutants displayed different phenotypes with respect to the diploid-specific hypersensitivity to DOX. While the diploid *tho2Δ* mutant was hypersensitive (>125-fold greater than WT) to DOX, the isogenic haploid *tho2Δ* mutant displayed only a modest (5-fold greater than WT) DOX sensitivity. However, both the haploid and diploid *hpr1Δ* mutants were hypersensitive (>125-fold greater than WT) to DOX (Table 2). We therefore examined the relative DOX sensitivity of isogenic haploid and diploid derivatives of various deletion mutants (*mft1Δ, thp1Δ* and *thp2Δ*) that encode other putative components of the THO complex that participate in mitotic recombination [49], [50]. Of these three additional THO associated mutants, only *thp1Δ* was detected in the initial diploid DOX screen (Table 1, Supplemental Table S1). Similar to the *tho2Δ* mutant, all three diploid deletion strains (*mft1Δ, thp1Δ* and *thp2Δ*) demonstrated enhanced hypersensitivity to DOX when compared to that in the isogenic haploid derivative (Table 2). Similar levels of sensitivity to HU and MMS were observed for the haploid and diploid derivatives of *mft1Δ* and *thp1Δ* when compared to WT. Although the haploid and diploid derivatives of the *thp2Δ* mutant showed similar levels of sensitivity to HU (5-fold greater than WT), the diploid derivative of the *thp2Δ* mutant showed enhanced hypersensitivity to MMS (25-fold greater than WT) compared to the haploid derivative which was not MMS sensitive (Table 2). Thus, for the majority of THO complex mutants, the diploid deletions demonstrate enhanced DOX sensitivity when compared to the isogenic haploid derivatives.

Since mating type transcription regulation alters relative expression levels and useage of DSB repair pathways (homologous recombination *versus* NHEJ), we examined whether the a1 α2 transcriptional regulators (from *MAT*) constitutively expressed from a selectable plasmid (pCB115) could reinstate sensitivity to DOX in the haploid deletion strains that showed sensitivity only when the deletion was established in the isogenic diploid strain. This plasmid has been previously characterized and confers a nonmating (diploid) phenotype in haploid BY4741 and BY4742 cells [41]. When the plasmid pCB115 was established in haploid MATα and/or MATa *bem1Δ, ctf4Δ, ctk1Δ, hfi1Δ, nup133Δ* or *tho2Δ* derivatives and these were exposed to DOX (25 or 50 ug/ml) in YPD agar, no increased sensitivity was detected when compared to the same strains containing the empty plasmid pRS315 (data not shown). This suggests that it is the diploid state and the availability of an additional recombinogenic chromosome (2n) rather than the diploid *MAT* expression pattern which is required to confer sensitivity to DOX in the diploid deletion strains.

### Diploid-specific doxorubicin sensitive mutants have altered mating type expression

It has been established that in diploid cells which exhibit altered *MAT* expression patterns, sensitivity to DSB damage is increased due to a decrease in recombination capability [51]–[53]. Moreover, many diploid deletion strains that exhibited IR hypersensitivity as a diploid showed less sensitivity to IR as haploids [40]. The enhanced sensitivity of some diploid deletion strains to DOX when compared to the isogenic haploid strain may indicate that alterations in mating type expression patterns in the diploid may be responsible for the enhanced lethality. To examine this possibility, we mated the diploid deletion strains that displayed diploid-specific DOX hypersensitivity (*tho2Δ, bem1Δ, nup133Δ, ctf4Δ, ctk1Δ* and *hfi1Δ*) to *MAT*a and *MAT*α mating-type tester strains to determine if mating in these diploid deletion strains was enhanced when compared to the non-mating WT diploid strain (Fig. 2B). Following individual mass matings of the diploid deletion strains with haploid *MAT*a and *MAT*α strains, we observed aberrant mating with either the *MAT*α tester strain (*ctk1Δ, hfi1Δ* and *nup133Δ*) or both the *MAT*a and *MAT*α tester strains (*ctf4Δ*). Only the diploid *tho2Δ* and *bem1Δ* strains showed non-mating similar to that observed with the WT BY4743 diploid strain (Fig. 2B). Since mass mating in patches does not allow determination of the number of cells that have converted into cells capable of mating, we streaked out the diploid deletion strains to obtain single colonies and tested these individual colonies for mating ability. Using this approach we determined that only in the case of the diploid *hfiΔ* mutant were all of the colonies completely converted to a *MATa* mating type phenotype. For the diploid *ctk1Δ* and *nup133Δ* mutants, most colonies (∼93%) demonstrated a higher rate of conversion to a *MATa* mating phenotype (*i.e.* mated colonies were “speckled” with small subsets of cells within colonies capable of growing on minimal medium as compared to WT colonies which were “non-speckled”) with very infrequent conversions towards the *MATα* mating phenotype and only some single colony isolates (2 and 7% respectively) were completely converted to a *MATa* mating phenotype. As expected for the diploid *ctf4Δ* mutant which demonstrates a chromosome loss phenotype, all single isolate colonies demonstrated a high rate of mating to either the *MATa* or *MATα* tester strains suggesting that either copy of chromosome III can be lost due to the high rate of malsegregation previously observed in these mutant cells. However, few of the single colony isolates from the diploid *ctf4Δ* strain were totally converted to either a *MAT*a or *MAT*α phenotype (2 and 1% respectively) suggesting that for the majority of cells mating type was unaffected and it is the loss of *CTR4* that is responsible for DOX sensitivity in this strain.

### Deletion of diploid-specific DOX resistance genes confers G1/S phase associated cell cycle progression defects

**Figure 3:**
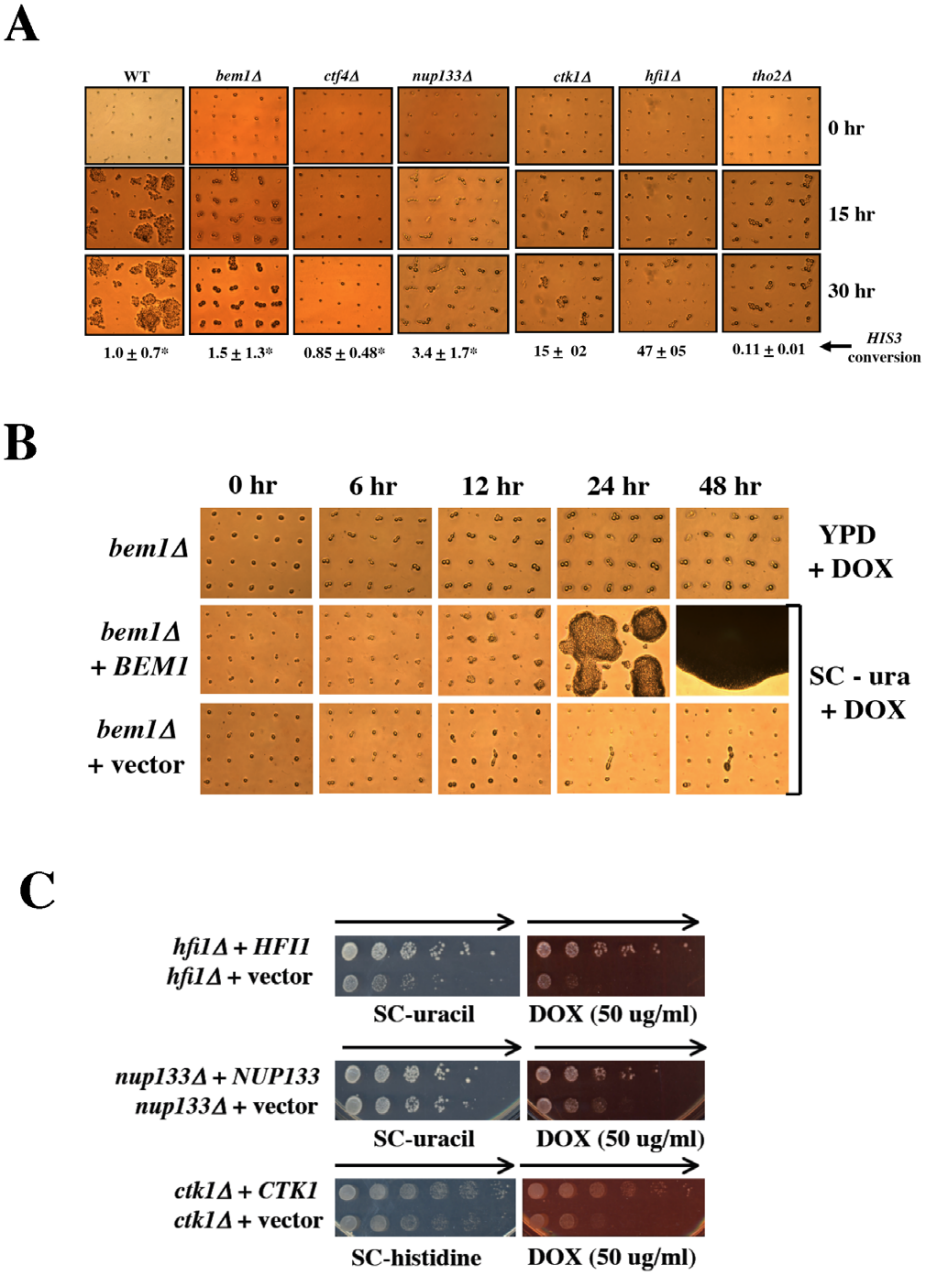
Deletion of diploid-specific doxorubicin resistance genes result in cell cycle progression or recombination defects. (A) WT and mutant diploid deletion strains were grown to logarithmic phase in liquid YPD. Single unbudded (G1) cells were arrayed into 5×4 cell grids on YPD with and without doxorubicin (50 µg/ml). Representative photomicrographs of mutant cells arrested in G1 or at G1/S following exposure to doxorubicin have been shown following 15 or 30 hr growth at 30°C. Only WT diploid cells were capable of forming viable microcolonies when exposed to doxorubicin. Most unbudded cells (>70%) from the WT and mutant diploid strains demonstrated rapid cell cycle progression and microcolony formation in the absence of DOX (data not shown). The mean gene conversion frequency of the *his3Δ1* allele to HIS^+^ was determined in WT and mutant diploid strains following transformation of a PCR fragment capable of restoring the *HIS3* allele following recombination. Conversion frequencies for the WT, *bem1Δ, ctf4Δ* and *nup133Δ* strains (*) have been previously reported [40]. The *HIS3* conversion frequencies for the diploid *ctk1Δ, hfi1Δ* and *tho2Δ* strains are the mean of 3–10 replica experiments±1 standard deviation. (B) Expression of *BEM1* within the diploid *bem1Δ* strain suppresses cell cycle arrest in G1 and restores viability following exposure to doxorubicin. The diploid *bem1Δ* strain was transformed with either empty vector or plasmid DLB1974 expressing the WT *BEM1* gene. Unbudded cells from the diploid *bem1Δ* strain with or without plasmid were grown as described above in liquid YPD or synthetic complete glucose containing medium lacking uracil (SC-ura) to maintain plasmid selection. Single unbudded cells were exposed to doxorubicin (50 µg/ml) in either synthetic complete glucose containing agar medium lacking uracil (SC-ura+DOX) to maintain the plasmid or YPD+DOX (for cells not containing plasmid). The *bem1Δ* cells exposed to DOX on YPD agar plates progress from G1 into S phase and arrest as budded cells (upper panels). Cells that harbor the *BEM1* expression plasmid (*bem1Δ*+*BEM1*) do not arrest in G1 or G1/S but form viable microcolonies by 24 hrs that continue to grow in the presence of DOX (middle panels). Diploid *bem1Δ* cells containing vector alone, arrest in G1 when exposed to DOX on SC-ura agar medium (bottom panels). (C) Expression plasmids containing *HFI1, NUP133* and *CTK1* suppress doxorubicin-induced lethality in the corresponding diploid deletion strains. Galactose-inducible expression constructs for *HFI1* and *NUP133* cloned within the selectable (*URA3*) plasmid BG1805 or vector alone were transformed into *hfi1Δ* and *nup133Δ* diploid strains respectively. The selectable (*HIS3*) plasmid containing *CTK1* and empty vector have been previously described [59]. Plasmid bearing cells were grown overnight at 30°C in either liquid SC-uracil containing galactose (for *hfi1Δ* and *nup133Δ* plasmid bearing strains) or SC-histidine glucose containing medium (for the *ctk1Δ* plasmid bearing strains) in 96 well dishes. Following serial 5-fold dilution, aliquots of each cell dilution were plated to the corresponding solid dropout medium with and without doxorubicin at the indicated concentration. Plates were photographed following 3 days growth at 30°C. In all cases, the expression plasmid restored resistance to DOX-induced lethality in the appropriate deletion strain. Arrows indicate direction of decreasing cell concentration.

The identification of DOX sensitive gene deletions that are diploid-specific suggests that these genes may mediate repair functions prior to the completion of DNA replication. Functionally, these genes may impact recombinational repair of DOX-induced lesions or alternatively, they may affect cell cycle progression (checkpoint) in G1 or early S phase. For those mutants that have defects affecting DNA damage checkpoint response, they may fail to elicit checkpoint arrest and continue to progress rapidly in the presence of damage (similar to the *rad9Δ* strain, data not shown) to produce inviable microcolonies. Alternatively, cells may not be able to re-enter the cell cycle (checkpoint adaptation or recovery defect) following cell cycle arrest and subsequent repair of DOX-induced DNA damage that occurs in G1 or early S phase. These cells demonstrate a prolonged arrest and fail to progress even after the repair of DNA damage (similar to *ccr4Δ* cells). We therefore examined the cell cycle progression of unbudded (G1) cells exposed to DOX for the six deletion strains that have diploid-specific sensitivity to DOX (Fig. 3). Following exposure to DOX for 15 or 30 hours, all of the diploid deletion strains examined (*bem1Δ, ctf4Δ, ctk1Δ, hfi1Δ, nup133Δ,*and,*tho2Δ)* demonstrated severe cell cycle progression defects when compared to the isogenic diploid WT strain. As described above (Fig. 1B) following exposure of WT diploid cells to DOX in G1, 60% (12/20) of the cells produced viable microcolonies that continued to grow into macrocolonies (Fig. 3A). However, for each of the deletion strains that were hypersensitive to DOX as diploids, cell cycle progression during G1 or following G1/S transition was severely inhibited following exposure to DOX (Fig. 3A). Mutant cells exposed to DOX in G1 demonstrated either prolonged arrest in G1 (*ctf4Δ*), arrested predominantly as budded cells following G1/S transition (*bem1Δ, ctk1Δ, hfi1Δ* and *tho2Δ)* or progressed to form a mixture of budded cells and small inviable microcolonies (*nup133Δ*). All of the mutants that progressed from G1 into S phase and arrested as budded cells or produced microcolonies exhibited cellular lysis. For some mutants (*nup133Δ*, *ctk1Δ* and *hfi1Δ*), this was evident at 15–30 hours following exposure to DOX (Fig. 3) while for the others (*bem1Δ, nup133Δ* and *tho2Δ*) lysis occurred at 48–96 hours following exposure (data not shown). Cell lysis following G1 to S phase transition and arrest as large budded cells was similar to that observed for *ccr4Δ* or *pop2Δ* mutants exposed to DOX or HU (Fig. 1B and [39]). Furthermore, following exposure to DOX, the majority of diploid *bem1Δ* mutant cells arrested as budded cells and most cells exhibited cellular enlargement (swelling). Similarly, an increase in cell size was observed for a smaller fraction of the *ctk1Δ* mutant cells exposed to DOX (Fig. 3A). The G1/S phase transition defects associated with these mutants and sensitivity to the S phase specific agents HU and MMS (Fig. 2) suggest that similar to cells with defects in the CCR4 damage response pathway, these mutants are unable to tolerate DOX-induced damage that induces replication stress. This apparently persists to elicit a prolonged S phase arrest and subsequent cell lysis.

Strikingly, the diploid *ctf4Δ* mutant exhibited a prolonged G1 arrest following exposure (Fig. 3A). These mutant cells failed to progress beyond the G1 phase of the cell cycle after extended time periods (96 hours exposure to DOX) even though most single unbudded cells (70%) were viable when these cells were arrayed onto YPD medium without DOX (data not shown). Although diploid *ctf4Δ* mutants appear to be competent for spontaneous recombinational repair [40], (Fig. 3A) damage-induced recombination may be compromised [54] suggesting that diploid *ctf4Δ* mutants may have persistent DOX-induced DNA damage that elicits a prolonged G1 arrest signal that prevents cell cycle progression similar to that seen for *rad51Δ* cells at high DOX doses (Fig. 1C). These results suggest that mutants which demonstrate diploid specific sensitivity to DOX have defects in recombination and/or checkpoint functions which severely inhibit cell cycle progression in G1 or during G1/S transition.

To confirm that the severe cell cycle defects and diploid-specific hypersensitivity to DOX was due to the observed diploid mutations, we complemented the *bem1Δ, ctk1Δ, nup133Δ* and *hfi1Δ* diploid deletion mutations with plasmids expressing the corresponding wild type gene (Fig. 3B,C). When the wild type *BEM1* gene was expressed in the diploid *bem1Δ* mutant strain, cell cycle arrest induced by DOX was abrogated, and many cells (50%) progressed rapidly through the cell cycle to form viable microcolonies (Fig. 3B). The DOX-induced cell cycle response of these cells was virtually identical to that observed with the WT diploid strain (Fig. 3A) and suggests that the *bem1Δ* is responsible for the severe cell cycle arrest phenomenon. The isogenic *bem1Δ* cells containing an empty plasmid arrested predominantly in G1 following exposure to DOX and most (75%) failed to progress into S phase. These cells subsequently lysed following prolonged exposure to DOX. Interestingly, unlike the *bem1Δ* cells exposed to DOX on synthetic complete (SC) medium the majority of single unbudded *bem1Δ* cells (75%) exposed to DOX in rich medium (YPD) rapidly progressed into S phase and arrested as large budded cells. These results suggest that in SC medium the cell cycle arrest response of *bem1Δ* cells to DOX is more rapid than that observed in YPD resulting in a clear G1 arrest. Moreover, the *BEM1* expression plasmid suppressed the enlarged cell size phenotype associated with the diploid *bem1Δ* strain (Fig 3B). Using a dilution plating assay, the DOX hypersensitivity of the diploid *hfi1Δ, nup133Δ* and *ctk1Δ* mutations was clearly suppressed when the corresponding wild type gene was expressed from a selectable plasmid (Fig. 3C). These results indicate that the identified gene deletions (and not an acquired second site mutation) are responsible for the diploid-specific hypersensitivity to DOX.

### The diploid-specific doxorubicin resistance genes *CTK1, HFI1* and *THO2* are required for recombination

Similar to the effects of deleting members of the RAD52 recombinational repair genes, defects in recombination pathways may result in hypersensitivity to DOX. Furthermore, we have detected mating-type expression defects among mutants that show diploid-specific hypersensitivity to DOX suggesting these mutants may be decreased in their ability to undergo recombinational repair. We therefore examined the diploid-specific DOX sensitive mutants for spontaneous PCR-mediated gene conversion of the endogenous *his3Δ1* allele, a process which is defective in RAD52 group mutants [40]. As previously reported, for the IR sensitive diploid *bem1Δ, nup133Δ* and *ctf4Δ* mutants, PCR-mediated gene conversion was similar to that in WT suggesting these mutants are recombination repair proficient [40]. Upon reexamination of these deletion mutants gene conversion frequencies comparable to that in WT were again observed (1.1, 4.3 and 2.4 fold increases as compared to WT was observed for *bem1Δ, nup133Δ* and *ctf4Δ* mutants respectively). However, both the diploid *ctk1Δ* and the *hfi1Δ* mutants showed significantly enhanced levels of gene conversion (15 and 47 fold increases as compared to WT respectively; Fig. 3A). The enhanced levels of gene conversion observed for the diploid *ctk1Δ* and the *hfi1Δ* mutants were significantly greater than that observed for the WT and was similar to that observed for the hyper-recombination mutant *hpr1Δ* (*i.e.* a 24 fold increase as compared to WT [40]). Furthermore, similar to that observed in strains deleted for members of the RAD52 recombinational repair group of genes, gene conversion in the diploid *tho2Δ* mutant was decreased 10 fold when compared to WT (0.11 of that observed for WT, Fig. 3A). These results suggest that for these diploid deletion mutants, recombinational repair of DSB damage may be impaired resulting in the observed hypersensitivity to the lethal effects of DOX. Hpr1 and Tho2 are both components of the THO complex that couples transcription elongation to recombinational repair and defects in these genes exhibit hyper-recombination phenotypes in haploids. We therefore examined diploid-specific DOX sensitive deletion strains that were defective in other members of the THO complex (*mft1Δ* and *thp2Δ*, as well as the DOX sensitive mutant *thp1Δ,* Table2) to determine if gene conversion was significantly altered. We found that gene conversion in the THO associated mutant strains was also significantly reduced when compared to WT (0.15, 0.12 and 0.12 for *mft1Δ*, *thp2Δ* and *thp1Δ* respectively). Thus, similar to the response of RAD52 group mutants to DSB damage, defects in the recombinational repair of DOX-induced DNA damage could lead to persistent unrepaired DNA damage which elicits a prolonged damage-induced checkpoint activating signal to mediate the lethal cell cycle arrest in G1 and/or following G1/S transition (Fig. 3).

Loss of *CTK1* function has been associated with contraction of the directly repeated rDNA sequences. Furthermore, since *CTK1* has been linked genetically to multiple IR and DOX resistance genes implicated in recombination repair (i.e., *RAD50, XRS2, MRE11, RAD51, RAD52, RAD54* and *RAD55*; [55]), as well as the THO complex component *MFT1* [56], we further examined the ability of haploid *ctk1Δ* mutants to tolerate integration of plasmid p306A2 at the *ADE2* locus which results in directly repeated *ade2* and a red colony phenotype. Direct integration in WT haploid strains (BY4741) and selection for the *URA3* marker results in yeast colonies that are red following establishment of the selectable marker. Establishment of the same plasmid marker following transformation into haploid *ctk1Δ* (or *hfi1Δ)* strains in the BY4741 (*MAT*a) haploid background resulted in colonies that were predominantly white suggesting that the hyper-recombination phenotypes associated with deletion of *CTK1* or *HFI1* would not tolerate the directly repeated *ade2* sequences (data not shown).

### Identification of an interactive genomic network defined by diploid-specific doxorubicin resistance genes

Using previously identified genetic and physical interactions compiled at SGD, we determined the interaction network for the 9 genes (*BEM1, CTF4, CTK1, HFI1, MFT1, NUP133, THO2, THP1* and *THP2*) which demonstrated diploid-specific sensitivity to DOX. We retrieved genetic and physical interaction data sets in Cytoscape v2.6.1 format. This query produced an initial genetic interaction network map with 502 nodes (genes) and 1075 edges (interactions) and a physical interaction map containing 188 nodes with 314 edges (data not shown). We subsequently combined the genetic and physical interaction maps and manually subtracted essential genes that were not found in the diploid deletion collection. Upon this map, we superimposed the DOX resistance genes identified in this study.

**Figure 4:**
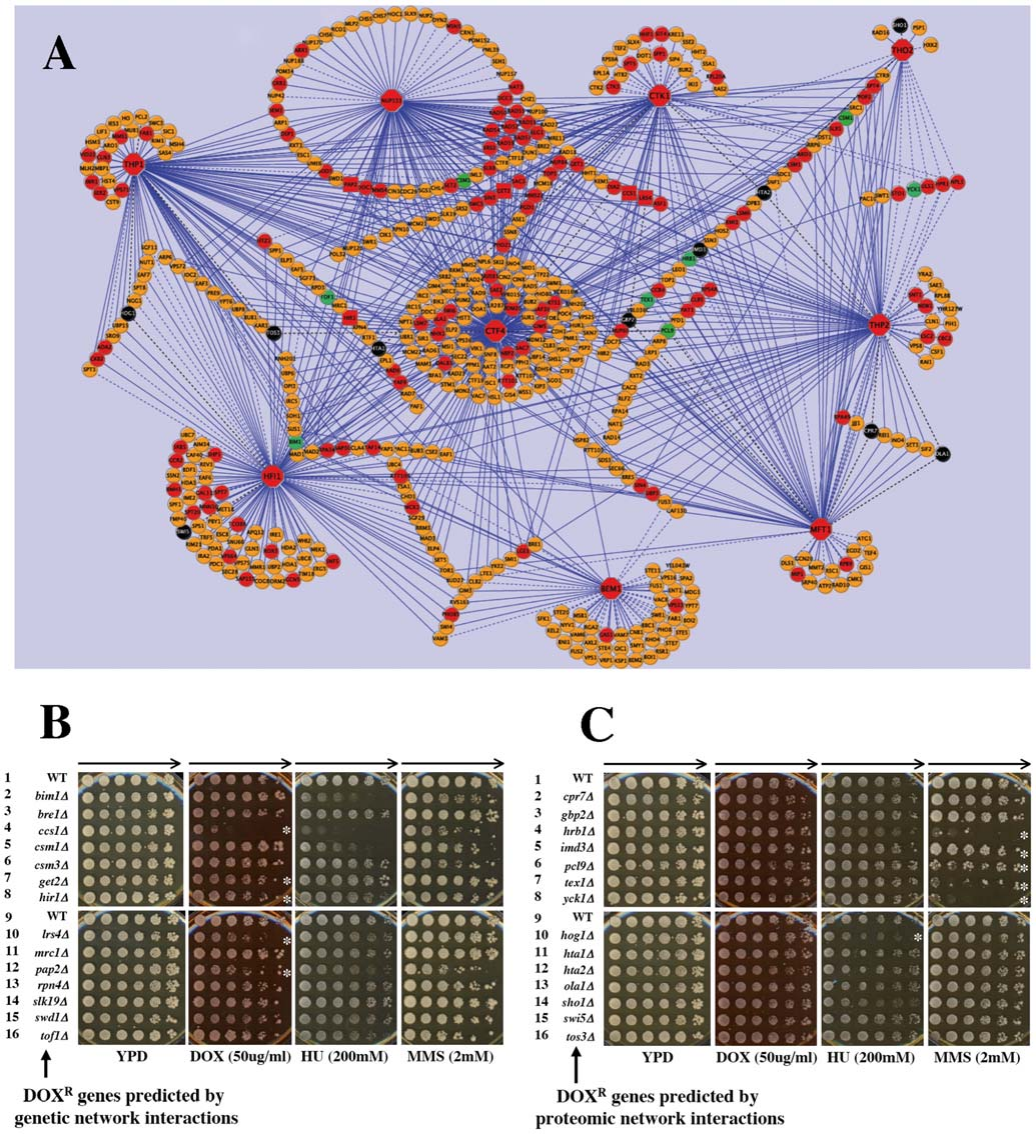
Genetic and proteomic interaction network of doxorubicin resistance genes identifies additional DNA damage resistance genes. (A) Using the 9 diploid-specific DOX resistance genes identified in this study (*BEM1, CTF4, CTK1, HFI1, NUP133, MFT1, THO2, THP1* and *THP2*; red octagon symbols), genetic and proteomic interactions were batch downloaded from data annotated at SGD as of Nov. 2, 2008. Genetic and physical interaction data sets were retrieved and visualized using Cytoscape v2.6.1. This initial genetic interaction network map contained a total of 502 nodes (genes) and 1075 edges (interactions) and the physical interaction map contained 188 nodes with 314 edges (data not shown). These were combined and all essential genes (*i.e.* deletions not represented in the diploid deletion collection) were eliminated resulting in a final combined interaction map with 500 nodes and 1154 edges. Genetic and proteomic interactions are indicated with a solid or dashed line respectively. Nodes (genes) that were identified in the initial diploid screen as conferring DOX resistance are denoted as red circles. Using the interactive genetic map as a predictive tool, additional DOX-resistance genes (red squares) were subsequently identified (see panel B). Some interactive genes/proteins (green circles) did not confer resistance to DOX but did confer resistance to other DNA damaging agents (HU and/or MMS, see panels B and C). Other gene deletion strains examined (black circles) did not show sensitivity to any of the damage agent tested when compared to WT. The diploid gene deletions associated with the remaining nodes (orange circles) were not tested for enhanced sensitivity to DNA damaging agents. (B) Identification of additional damage resistance genes based on genetic interactions with diploid-specific DOX resistance genes. Fourteen diploid deletion strains predicted to be DOX sensitive based on genetic interactions (rows 2–8 and 10–16) were obtained from the diploid deletion collection and tested for enhanced sensitivity to DOX, HU and MMS when compared to WT. Cell growth, dilution and replica plating techniques were as described in Fig. 1A. Some strains showed enhanced sensitivity to DOX (*ccs1Δ*, row 4; *get2Δ*, row 7; *hir1Δ*, row 8; *lrs4Δ*, row10 and *pap2Δ*, row12) when compared to WT (rows 1 and 9). These strains demonstrate modest (5-fold; *get2Δ* and *hir1Δ*) to moderate (25–125 fold; *ccs1Δ, lrs4Δ* and *pap2Δ*) enhanced sensitivity to DOX as indicated (*). Some strains showed enhanced sensitivity (5–125 fold) to HU (*bim1Δ*, row 2 and *csm1Δ*, row5) or MMS (*tof1Δ*, row 16) without accompanying sensitivity to DOX. (C) Identification of additional damage resistance genes based on proteomic interactions with diploid-specific DOX resistance genes/proteins. Diploid deletion strains predicted to be DOX sensitive based on proteomic interaction map were obtained from the diploid deletion collection and tested for enhanced sensitivity to DOX, HU and MMS when compared to WT. Cell growth, dilution and replica plating techniques were as described in Fig. 1A. None of the deletion strains were found to show enhanced sensitivity to DOX. However, some strains (*hrb1Δ*, row 4; *imd3Δ*, row 5; *pcl9Δ*, row 6; *tex1Δ*, row7; and *yck1Δ*, row 8) showed enhanced sensitivity to MMS (5–625 fold) and *hog1Δ* (row 10) showed enhanced sensitivity to HU (5 fold) when compared to WT (rows 1 and 9) as indicated (*).

The resulting union of the genetic and physical interaction maps produced a combined map that was defined by 500 nodes with 1157 interconnected direct interactions (Fig. 4A). Within this combined diploid-specific G1 repair network, nine highly interactive gene nodes acted as “hubs” to directly interconnect as first neighbors with seven of the other “hub” genes important for the diploid-specific toleration of DOX damage. Thus, of the nine diploid-specific DOX resistance genes, *MFT1* was the most interactive major hub member connecting to four other major hubs (*NUP133, CTK1, THO2* and *THP2*). When considering first neighbor interactions, the most highly interactive of the major hubs were the diploid-specific DOX resistance genes *CTF4, HFI1, THP1*, *NUP133, CTK1* and *THP2* which, interacted directly with 227, 121, 112, 104, 86 and 85 other gene nodes as first neighbors respectively (Fig. 4A). Within this combined genetic and physical interaction network we identified a total of 123 (32.7%) of the 376 DOX resistance genes found in this study suggesting that a diploid-specific DOX resistance gene network may be a significant fraction of the total genes identified which mediate resistance to DOX. Significantly, most members of the RAD52 recombinational repair group that are DOX resistance genes including *RAD50, RAD51, RAD52, RAD54, RAD55, RAD57, RAD59* and *XRS2*) were all found to interact jointly as a cluster with *CTK1, NUP133* and *CTF4*. Within this interactive cluster are other diploid DOX resistance genes that have been implicated in DSB repair including *DCC1, ELG1* and *ASF1.*

Within the combined genetic/proteomic interaction map are 377 gene nodes that interconnect with the major diploid-specific DOX resistance gene node hubs yet were not detected in the DOX screen. Since many of these genes display genetic interconnectivity with multiple major DOX resistance gene nodes in a pattern similar to that for other DOX resistance genes identified in the screen, we examined 14 of these genetically predicted and multiply interconnected diploid deletion strains for sensitivity to DOX, HU and/or MMS. Of the 14 mutants examined that interconnect to multiple major DOX resistant gene nodes (*bim1Δ*, 6 nodes; *bre1Δ*, 4 nodes; *ccs1Δ*, 2 nodes; *csm1Δ*, 4 nodes; *csm3Δ*, 2 nodes; *get2Δ*, 3 nodes; *hir1Δ*, 4 nodes; *lrs4Δ*, 2 nodes; *mrc1Δ*, 3 nodes; *pap2Δ*, 2 nodes; *rpn4Δ*, 4 nodes; *slk19Δ*, 3 nodes; *swd1Δ*, 4 nodes and *tof1Δ*, 3 nodes) five mutants (*ccs1Δ, get2Δ, hir1Δ, lrs4Δ* and *pap2Δ*) were found to express enhanced DOX sensitivity to varying degrees (5–125 fold) when compared to WT (Fig. 4B). When compared to WT, some mutants showed enhanced sensitivity to HU and/or MMS without accompanying sensitivity to DOX (*bim1Δ, csm1Δ, csm3Δ* and *tof1Δ*) and some (*bre1Δ, mrc1Δ, rpn4Δ, slk19Δ* and *swd1Δ)* showed no sensitivity to any of the DNA damaging agents tested (Fig. 4B). Thus the genetic interaction network map was capable of identifying additional mutations that impact on resistance to DNA damaging agents including DOX.

In a similar manner we utilized the proteomic interaction map within Fig. 4A to identify potential DOX sensitive diploid deletion mutants not detected in the initial screen. We examined 14 diploid mutants not detected in the initial DOX screen that interconnect to DOX resistance gene nodes defined within the proteomic network (Fig 4A). Of the 14 diploid deletion mutants examined which interconnect to DOX resistance gene nodes (*cpr7Δ*, 2 nodes; *gbp2Δ*, 3 nodes; *hrb1Δ*, 4 nodes; *imd3Δ*, 2 nodes; *pcl9Δ*, 2 nodes; *tex1Δ*, 3 nodes; *yck1Δ*, 2 nodes; *hog1Δ*, 2 nodes; *hta1Δ*, 1 node; *hta2Δ*, 2 nodes; *ola1Δ*, 2 nodes; *sho1Δ*, 1 node; *swi5Δ*, 1 node and *tos3Δ*, 2 nodes), none were found to be DOX sensitive when compared to WT (Fig. 4C). However, five diploid mutants (*hrb1Δ, imd3Δ, pcl9Δ, tex1Δ* and *yck1Δ*) were found to exhibit enhanced sensitivity to MMS (5 to >625 fold) when compared to WT and one mutant (*hog1Δ*) displayed enhanced (5 fold) sensitivity to HU (Fig. 4B). These results indicate that the interactive proteomic network defined by previously identified diploid-specific DOX sensitive mutants is less predictive than genetic interconnectivity in identifying additional DOX resistance genes. Thus, construction of interactive gene networks similar to those described here (Fig. 4A) is a valuable tool for gene discovery and suggests that further damage response genes not detected during primary screening remain to be identified and characterized from within this interaction map.

## Discussion

Large sets of novel genes that mediate resistance to a variety of DNA damaging agents have been identified using the isogenic yeast deletion strain collections (reviewed in [57]). Surprisingly, screens previously considered near saturation by classical mutagenesis screening methods, including those for ionizing radiation (IR) sensitivity [58], have uncovered a large number of previously uncharacterized radiation resistance genes [40], [39]. The fact that some of these were the first genome-wide radiation screens performed in diploid cells accounts in part for the discovery of such a formidable list of new radiation resistance genes since these screens take advantage of a novel aspect of yeast repair biology. Yeast have a compact, non-redundant genome with few repeated genes or repetitive DNA sequences. This promotes IR-induced DSB damage to be preferentially repaired by homologous recombination which requires an undamaged homolog or sister chromatid to template a successful repair event. Haploid yeast cells lack a homolog in G1 or early S phase, where sister chromatids may only be partially replicated. Therefore, in unsynchronized haploid cells that have been irradiated throughout the cell cycle, as radiation dose increases, a rapid dose-dependent decline in survival is observed followed by a more gradual radioresistant decline in survival. This two-component survival response has been attributed to the exquisite radiosensitivity of haploid cells in G1 where no homolog is available to template a successful recombinational repair event. Under these circumstances in G1 cells, one DSB “hit” is lethal. The second, radio-resistant repair component is thought to reflect the capability of cells in late S and G2 phases to repair IR-induced DSBs by recombination. Since diploid mutants have a chromosome homolog in G1, they are radioresistant throughout the cell cycle and thus facilitate the detection of previously unknown DSB repair gene mutants that impact checkpoint and/or recombinational repair functions in G1 or early S phase prior to the completion of DNA synthesis.

Our diploid screen identified 376 gene deletions sensitive to the DNA damaging agent doxorubicin (DOX), many of which overlap with those that function in recombinational repair and were also identified in our previous diploid IR screens (Table 1). However, when a similar screen was performed in the isogenic haploid deletion collection, far fewer haploid mutants (71) were detected that mediated resistance to DOX [38]. We can attribute our enhanced success at identifying DOX resistance genes to two factors. First, our diploid screen employed DOX doses that were greater than that used in the haploid screen. Secondly, DSB damage such as that induced by DOX or IR is repaired preferentially by recombinational repair mechanisms utilizing a homologous chromosome or sister chromatid. DNA damage resistance genes that confer resistance to DOX and function exclusively in G1 or at the G1/S boundary prior to DNA synthesis are undetectable as mutants in haploid cells which have no homolog capable of serving as a template for recombination repair prior to DNA replication. The fact that many of these DOX resistant genes also overlap with mutants within a BRCA1 suppressor pathway that regulates transcription elongation, RNA polymerase II stability as well as mRNA export and decay in G1 [59], mutants that affect cell size control in G1 [43], [44], mutants sensitive to oxidative damaging agents that cause damage in G1 [60], [45] or mutants hypersensitive to the G1-specific toxin zymocin (Table 1) adds further support to the identity of G1-specific repair processes in which RNA metabolism may play a critical role in resistance to DSB damage.

Nine diploid specific DOX resistance genes were identified and appear to be functionally interrelated as numerous genetic and proteomic interactions have been documented for these genes (Fig. 4A). Functions for these genes are diverse however, all have previously described, repair-related phenotypes. For example, defects in *CTK1, CTF4, NUP133* and members of the THO complex all have been previously implicated in mediating repair responses to DNA damage [61]–[63], [54]. Furthermore, deletion of *BEM1, CTF4*, *HFI1* and *NUP133* were previously identified as IR resistance genes (Supplementary Table S1 [40];) and the *hfi1Δ* was detected in the haploid DOX screen [38] although in our hands, the sensitivity to DOX in the *MAT*a haploid (BY4741 strain background) was minimal at best and the isogenic *MAT*α *hfi1Δ* derivative displayed no sensitivity to DOX (Fig. 2B).

Detailed examination of the cell cycle progression of single, unbudded G1 cells for six of these mutants clearly demonstrated that all have cell cycle progression defects associated with G1 or G1/S phase transition. However, since defects in recombination repair could promote the persistence of DSB damage and cause extended cell cycle delays, we examined these six mutants for the ability to undergo PCR mediated gene conversion of the *his3Δ1* allele by homologous recombination. As previously reported [40], for three diploid mutants that were also IR sensitive, (*bem1Δ, ctf4Δ* and *nup133Δ*), no defect in spontaneous recombination could be identified when compared to WT suggesting that, with the exception of the *ctf4Δ* strain that appears to be specifically deficient for damage-induced recombination [54], the *bem1Δ* and *nup133Δ* mutants can be provisionally classed as checkpoint defective. For two mutants, (*ctk1Δ* and *hfi1Δ)* spontaneous recombination at *his3Δ1* was significantly elevated when compared to WT, while for the diploid *tho2Δ*, gene conversion was significantly decreased. Moreover, other THO-associated mutants that displayed enhanced sensitivity to DOX as diploids also demonstrated significantly decreased levels of recombination as assayed by gene conversion. Since this decrease in gene conversion was similar to that observed for RAD52 group mutants [40], this suggests that the hypersensitivity of these THO-associated diploid mutants to DOX may arise from defects in recombinational repair of DOX-induced DSBs. This hypo-recombination defect in gene conversion for some (*tho2Δ, mft1Δ, thp2Δ*) but not all (*hpr1Δ*; [40]) mutated members of the THO complex, is in contrast to the hyper-rec phenotypes described for THO defects when assayed by loss of stability in directly repeated sequences integrated at *LEU2* [49]. This discrepancy may reflect differences in the genetic requirement for recombination between directly repeated integrated DNA sequences which involves single-strand annealing *versus* that for gene conversion [64], [65] as assayed in this study by PCR-mediated restoration of *HIS3* following transformation.

The magnitude of the hyper-recombination phenotype for the *ctk1Δ* and *hfi1Δ* mutants was similar to that described for the diploid *hpr1Δ* mutant [40] which has been extensively characterized as expressing a hyper-rec phenotype for mitotic recombination events [66], [67] and functions in transcription elongation [68] further linking transcription to recombination. Therefore, it is reasonable to suggest a model in which Ctk1, as part of the CTDK-I kinase complex that phosphorylates the RNA polymerase II C-terminal domain and facilitates transcription elongation [69], [70], also participates in recombination. Ctk1 is also required for BRCA1-induced lethality in yeast through its participation in an mRNA export/decay pathway [59]. Mutants in this pathway which suppress BRCT-induced lethality in yeast, all exhibit sensitivity to DNA damaging agents. These results suggest that Ctk1 and the CTDK-I kinase complex may contribute indirectly to, or alternatively, participate directly in transcription associated recombination (TAR) in which Hpr1 and other members of the THO complex as well as the Rad52 group of repair genes are required for recombination between direct repeated sequences [71], [72]. Consistent with this idea is the finding that directly repeated rDNA sequences undergo contraction in *ctk1* mutant strains [73] suggesting a hyper-rec phenotype associated with directly repeated DNA sequences. Our finding that directly repeated *ade2* sequences are not stable in *ctk1Δ* (data not shown) further implicates *CTK1* as a gene required for the maintenance of directly repeated genomic sequences. It is also possible that loss of CTDK-I function may promote RNAPII “stalling” during transcription elongation and similar to other THO mutants that interfere with transcription elongation, promote the formation of recombinogenic DNA:RNA hybrids [74]. Such structures may interfere with replication fork progression [75] suggesting a possible mechanism for cell progression defects that extend into S phase in these mutants when exposed to DOX. Finally, deletion of *CTK1* has been found to be synthetically lethal when combined with deletions in genes required for TAR including those involved in DSB recombination such as *RAD50, RAD51, RAD52, RAD54* and *RAD55* [55] as well as *MFT1* [56], a key component of the THO complex. Taken together, these results suggest that CTDK-I transcription elongation functions may be critical for the formation of TAR complexes.

Although IR induced DSBs were predominantly described as arresting cells at G2/M, recent reports describing the genetic checkpoint controls associated with DNA damage occurring in G1 are accumulating. Evidence for recombinational repair of DSBs specifically in G1 is sparse due to the continued preference for using haploid yeast in checkpoint and DNA damage related studies. Furthermore, although it was originally thought that DSB resection, which is required for homologous recombination, did not occur during G1 in haploids [76], recent results suggest that radiation-induced DSBs are efficiently processed for homologous recombination in G1 haploids [77]. Since G1 haploids lack an undamaged homolog to template recombination repair, and NHEJ does not function efficiently on IR-induced DSBs, almost all IR-induced DSB lesions that occur in G1 are lethal. Moreover, DSB-induced checkpoint functions have been well documented in G1 haploids (see below) and are equally unlikely to enhance survival following DNA damage that induces DSBs in G1 haploids. Thus the sensing and processing of DSB damage by haploids in G1 may be an atavistic repair trait useful only in the parental diploid organism from which the haploid is derived following meiosis.

Damage induced arrest in G1 or at G1/S transition has not been as well characterized in *S. cerevisiae* since it is more transient than the arrest seen at G2/M [78]. A few early studies identified a G1 checkpoint in haploid yeast that was strongly activated by UV or MMS damage [79]–[81]. WT cells arrested by alpha factor in G1 (i.e. *CDC28* dependent cell cycle arrest at START) are delayed for a short period of time (∼20–40 minutes) in G1 prior to the onset of budding following UV irradiation. This short delay is not observed in isogenic *rad9Δ* cells [82]. A robust, *RAD9* and *RAD17* dependent G1 arrest following IR has been demonstrated in haploid cells continually irradiated at a low, sub-lethal dose [83]. In fact, G1 delays as long as 18 hours could be seen for WT cells given a single sub-lethal dose; this was not observed in *rad9rad17* double mutants [83]. More recent studies have identified a G1 damage checkpoint that requires the activity of Dot1, histone H3, and Rad9 [84] as well as Tel1 and H2A [85], [86]. In addition, the CDK Pho85 appears to be involved in G1 checkpoint adaptation [87]. To initiate damage-mediated G1 checkpoint arrest, Rad9 binds to H2A and/or H3 methylated by Dot1 and the ATM and ATR orthologs Tel1 and Mec1 activate Rad53p which phosphorylates Swi6p and inhibits *CLN1* and *CLN2* expression to delay cell cycle progression at G1/S [88]. Thus evidence for the genetic control of G1 arrest phenomena in response to DNA damage suggests that additional checkpoint associated genes such as those identified in this study may contribute to enhanced survival of diploids.

We have previously identified the CCR4 damage response network and the checkpoint associated roles of *CCR4* and *DHH1* whose protein products have nuclear functions and play key roles in mRNA decay at cytoplasmic P-bodies [40], [89], [39]. Characterization of the *ccr4Δ* and *dhh1Δ* mutants indicated that they were required for G1 and S phase cell cycle progression following radiation or replication stress. Furthermore, *CCR4* was found to be a member of the *RAD9* epistasis group of IR resistant checkpoint genes. Specifically, *CCR4* and *DHH1* behave as checkpoint adaptation genes since following IR, diploid *ccr4Δ* or *dhh1Δ* cells show prolonged arrest in G1; therefore they are required for re-entry into the cell cycle following DNA damage. Strikingly, *ccr4Δ* and *dhh1Δ* mutants are sensitive to IR only as diploids and not as haploids, indicating that the radiation sensitive IR defect lies in G1 [39]. Surprisingly, DOX-induced DNA damage appears to be equally lethal in isogenic diploid and haploid *ccr4Δ* cells and cell cycle progression of diploid *ccr4Δ* cells exposed to DOX in G1 is not delayed. Cells progress rapidly into S phase, arrest and undergo lysis similar to that observed for the diploid *bem1Δ, ctk1Δ, hfi1Δ, nup133Δ* and *tho2Δ* strains exposed to DOX in YPD. This suggests that there may be differences in the spectrum of DSB damage induced by IR and DOX. Moreover, the overlap of gene deletions that confer both DOX and IR sensitivity is 28% (Table 1) suggesting that a significant number of genes are specific for DOX-induced DNA lesions. Alternatively, the influx of DOX into *ccr4Δ* cells may be delayed or reduced due to sequestering of DOX through interaction within the rich YPD agar plate medium. A difference in the DOX-induced cell cycle arrest kinetics for diploid *bem1Δ* mutants plated to YPD as compared to synthetic complete medium suggests that cell cycle arrest is delayed until S phase in YPD compared to a G1 arrest observed on synthetic complete medium (Fig. 3B). Therefore, DOX-induced DSB damage may not occur until G1/S transition or early S phase in rich medium (YPD) and it would not be possible to elicit a checkpoint arrest response in early G1. Since a recombinational repair defect can also prolong cell cycle arrest, we determined that *ccr4Δ* cells were recombination proficient based on three recombination-related assays [39]. Both haploid and diploid *ccr4Δ* strains are sensitive to S phase specific DNA damaging agents such as HU, indicating that *CCR4* haploid and diploid mutants share a common checkpoint repair defect that extends into S phase. The checkpoint repair defects found in *ccr4Δ* and *dhh1Δ* diploid cells have subsequently been confirmed by other laboratories [90]–[93].

Since homozygosity at the mating-type locus in diploid yeast can decrease the resistance to DNA damage and especially DSBs, we investigated whether deletions of our diploid-specific DOX resistance genes could affect mating type expression in diploids. In some, (*ctk1Δ*, *nup133Δ*, *hfi1Δ* and *ctf4Δ*) but not all of the diploid specific DOX sensitive mutants, the gene defects did affect mating type expression in the diploid cells. Although both the diploid *ctk1Δ* and *nup133Δ* strains maintained a predominantly *MATa/MATα* phenotype, a much higher rate of conversion to *MATa* cells was observed when compared to the WT diploid. In the diploid *hfi1Δ* strain, all cells demonstrated complete conversion to the *MATa* mating phenotype while in the *ctf4Δ* mutant, high rates of conversion to both *MATa* or *MATα* was observed consistent with the described role of Ctf4 in maintaining chromosome stability and sister chromatid cohesion [94]. No elevated changes in mating-type expression were observed for either *bem1Δ* or *tho2Δ* diploid strains when compared to WT.

Repression of *MAT*a and *MAT*α combined with concomitant expression of diploid-specific genes affects radiation resistance. IR resistance is enhanced in diploid *MATa/MATα* cells compared to isogenic *MATa/MATa* or *MAT*α*/MAT*α diploid cells [51]–[53]. This effect can occur in haploids (through de-repression of silent mating type loci in *SIR* mutants) or in diploids and can suppress the effects of mutations in multiple recombination genes including *RAD51, RAD52* and *RAD55* [95], [53] or mutations in the post replication repair pathway [96], [97]. Almost complete repression of the NHEJ pathway also occurs when diploid-specific genes are expressed due to severe down-regulation of *NEJ1* [98]–[100]. Thus, in the absence of PRR, DSB damage appears to be preferentially channeled into *RAD52*-dependent recombination in *MAT*a/*MAT*α diploids by downregulating NHEJ. Moreover, *MAT*a/*MAT*α expression in a haploid also impacts checkpoint adaptation functions causing prolonged *RAD9*-dependent G2 arrest following a site-specific DSB [41]. Our finding that some DOX sensitive mutations (*i.e., ctk1Δ, nup133Δ* and *ctf4Δ*) are sensitive as diploids but not as haploids appears not to be the result of diploid specific expression of *MAT*a since the majority of cells maintain the non-mating *MATa/MATα* phenotype typical of repair proficient diploid strains. Instead, these mutants appear to be damage sensitive due to checkpoint or recombination defects conferred by the loss of the individual gene function(s). Conversely, the *hfiΔ* strain demonstrated complete conversion to a *MATa* mating phenotype, yet the *MAT*a diploid still displayed a hyper-rec gene conversion phenotype. This suggests that during strain propagation, high rates of gene conversion at *MAT* resulted in selection for a *MATa/MATa* diploid population yet still retained the capability for elevated levels of homologous recombination once the *MAT* conversion had occurred.

Using previously published genetic and proteomic interactions, we successfully predicted and identified new IR resistance genes based on interactions with members of the CCR4 damage response network [39]. A similar approach using the published genetic and proteomic interactions annotated within the SGD identified five DOX resistance genes that were not detected in the primary screen and exhibited an intermediate sensitivity to DOX induced cytotoxicity (Fig. 4). Interestingly, all of the DOX resistance genes successfully identified in this manner were predictions based on genetic but not proteomic interactions. The more robust genetic predictions used to identify these additional DOX resistance genes are based primarily on synthetic lethality or fitness interaction data [101], [102], [55]. The limitations of proteomic predictions for gene discovery and enhanced value of genetic as compared to proteomic interaction data has been previously noted [103]. Presumably, the robust nature of the genetic interaction data reflects the *in vivo* as opposed to the *in vitro* nature of proteomic determinations that tend to miss interactions with loosely associated proteins or misidentify interactions with overly abundant proteins [104]. Another nine diploid deletion strains predicted as being DOX sensitive showed little or no sensitivity to DOX but were sensitive to HU and/or MMS indicating that our diploid specific genes interact with other damage repair modules not required for the spectrum of DNA damage lesions induced by DOX. One DOX resistance gene identified in this manner is *LRS4* (*l*oss of *r*DNA *s*ilencing; [105]), exhibits synthetic lethal interactions with deletions of *CTK1* as well as *CTK2* and *CTK3*, (other members of the CTDK-I complex). Examination of the diploid *lrs4Δ* strain indicates that cells have a G1/S progression defect similar to that in the isogenic *ctk1Δ* diploid strain when exposed to DOX (data not shown). This suggests that Lrs4 may be a phosphorylation target of CTDK-I and this interaction may be required to suppress recombination at directly repeated sequences such as that found at the rDNA or *ade2-URA3-ade2* loci.

Our yeast screen identified 376 DOX resistance genes and the majority (76%) are conserved suggesting they may have clinical relevance. DOX is a highly effective anthracycline chemotherapeutic agent that targets solid tumors of the breast and other cancers; however, dosage has to be carefully regulated and monitored to avoid the potentially life threatening complications associated with cardiotoxicity. DOX is a DNA damaging agent that produces DNA DSBs in part through the production of reactive oxygen species (ROS). The site of ROS production appears to be the mitochondrion and yeast mutants that lack functional mitochondria are indeed more resistant to DOX (Fig. 1E). Cardiotoxicity appears to occur because DOX-induced ROS is excessive in mitochondria rich tissues such as the heart, resulting in respiratory failure and/or severe mitochondrial damage. This in turn results in cardiomyocyte cell death and subsequent cardiac failure. Of significance, is the fact that our screen is enriched for mutations (n = 30) in genes that are associated with mitochondrial functions, and most (29/30 = 96%) are highly conserved (Table S3). Presumably, these mutations may promote enhanced DOX mediated ROS production and/or allow greater access of DOX into the mitochondrial compartment. Mutations or polymorphisms in these genes within human populations may therefore predict cardiotoxicity due to enhance hypersensitivity of cardiac tissue to DOX.

DOX resistance in tumors can occur which decreases the efficacy of this chemotherapeutic agent. In some cases this can severely limit the clinical usage of this otherwise effective class of drugs. We propose that tumor hypersensitivity and/or resistance to DOX is genetically determined and that the orthologs identified in this study offer many new potential genes that could be targeted for inactivation to increase tumor sensitivity to DOX chemotherapy. Validating these human orthologs as genes which confer resistance to DOX could allow strategies to be designed that sensitize DOX resistant cancers that would be normally refractory to treatment with this drug. From our extensive list of highly conserved DOX resistance genes identified in yeast, we utilized BLAST analysis to identify five DOX resistance targets that show high homology to proteins previously identified to be mutated in breast cancer (Table S4; [106]). These targets may be predictive of an enhanced and more complete clinical response to lower doses of the drug. Recently, one of these predicted targets, PRPF4B (PRP-4) has been validated as a DOX resistance gene in ovarian cancer cells [107]. Moreover, these authors also identified and validated that a component of the human THO complex (THOC1) is a DOX resistance gene in human ovarian cells [107]. Of further significance is the finding that a human TREX component (hTREX84), which is required for transcription elongation and mRNA export is significantly overexpressed in human breast cancers [108]. Since the conserved THO/TREX complexes interact together in both yeast and human cells to link transcription elongation to mRNA export [109], these results suggest that tumors with THO/TREX expression abnormalities may be DOX hypersensitive. As THOC1 is the ortholog of yeast *HPR1* (Table 2), expression defects associated with the other THO complex orthologs (*THO2, MFT1* and *THP2*) are similarly predicted to confer DOX sensitivity in human cells. If expression or mutational defects in these genes can be identified, they may be important determinants for predicting an effective clinical response to DOX therapy.

## Supporting Information

Table S1 Yeast diploid deletion mutants hypersensitive to the lethal effects of doxorubicin with associated sensitivity to the toxin zymocin. Doxorubicin hypersensitivity in the diploid deletion strains was scored from 1–3 (complete description in Results section of text) with 1 being the least sensitive and 3 the most sensitive. Sensitivity to zymocin in the diploid deletion strains was scored 1–3 with 1 being the least sensitive and 3 being the most sensitive (see complete description in Results section of text). Diploid deletions that are cross sensitive to ionizing radiation (IR) have been indicated in bold. References that describe haploid deletion strains that are sensitive to doxorubicin, are defective in G1 cell size control and cross sensitive to oxidative damaging agents are described in the text (see Results section for detailed description). Human orthologs and associated P-values were determined by protein BLAST analysis. Yeast protein sequences were obtained from SGD and BLAST analysis was used to identify orthologs within the human reference protein database at NCBI. Gene functions and cellular component of corresponding yeast proteins were obtained from SGD.
(0.11 MB XLS)

Table S2 Yeast diploid deletion mutants that show reduced (slow) growth in response to doxorubicin. Table listing diploid yeast gene deletions that have a slow growth rate when exposed to doxorubicin. Deletion strains were scored 1–2 with 1 being the least inhibited and 2 being more inhibited when exposed to doxorubicin (see text Results section for complete description). Zymocin sensitivity of diploid deletion strains has been described in Table S1. Cross sensitivity to IR (bold) has been indicated for the diploid deletion strains. Cross sensitivity of haploid deletion strains to doxorubicin, G1 size control and oxidative damage are identical to that described in Table S1. Gene function, cellular component location of protein products, human orthologs and associated P-values are identical to that described in Table S1.
(0.10 MB XLS)

Table S3 Highly conserved mitochondrial gene targets that mediate doxorubicin resistance in diploid yeast. Table listing doxorubicin sensitive yeast diploid gene deletions with products implicated in mitochondrial function (see text Discussion section for complete description).
(0.06 MB DOC)

Table S4 Yeast doxorubicin resistance genes whose protein products are orthologs of human proteins encoded by genes mutated in breast cancer. Table listing yeast doxorubicin resistance proteins that are orthologs of human proteins encoded by genes found to be mutated in breast cancer (see text Discussion section for complete description).
(0.08 MB DOC)
