# Supplementary material for: Comparative Genome-Wide Screening Identifies a Conserved Doxorubicin Repair Network That Is Diploid Specific in Saccharomyces cerevisiae
Source: PLoS One. 2009 Jun 8;4(6):e5830. doi: 10.1371/journal.pone.0005830 (PMC2688081; doi:10.1371/journal.pone.0005830)
Supplement: Table S3 — Highly conserved mitochondrial gene targets that mediate doxorubicin resistance in diploid yeast. Table listing doxorubicin sensitive yeast diploid gene deletions with products implicated in mitochondrial function (see text Discussion section for complete description). (0.06 MB DOC) [file pone.0005830.s003.doc]

**Table S3.** **Highly conserved mitochondrial gene targets that mediate doxorubicin resistance in diploid yeast**.

| **Yeast gene deletion** | **Yeast Function^1^** | **Cellular Component^2^** | **Human Ortholog^3^** | **P-value** |
| --- | --- | --- | --- | --- |
| ACO1 | ***ACO***nitase: Aconitase: TCA cycle | mito | ACO2 | 0.00E+00 |
| ADK1 | ***AD***enylate ***K***inase | mito, cyto | AK2 | 3.00E-70 |
| DOA4 | ***D***egradation ***O***f ***A***lpha: Ubiquitin isopeptidase | mito, prot, memb | USP8 | 3.00E-51 |
| DOC1 | ***D***estruction ***O***f ***C***yclin B | mito | ANAPC10 | 2.00E-22 |
| ECM33 | ***E***xtra***C***ellular ***M***utant | mito, cell wall, memb | MUC21 | 9.00E-09 |
| ERG6 | ***ERG***osterol biosynthesis | mito, ER | TGS1 | 3.00E-05 |
| FUN12 | ***F***unction ***U***nknown ***N***ow: translation initiation | mito, cytosolic ribosome | EIF5B | 0.00E+00 |
| GET1 | ***G***olgi to ***E***R ***T***raffic: Subunit of the GET complex | ER, mito | none | none |
| IFM1 | ***I***nitiation ***F***actor for ***M***itochondrion translation | mito | MTIF2 | 2.00E-102 |
| KHA1 | ***K***+/***H***+ ***A***ntiporter: Putative K+/H+ antiporter | mito, golgi | TCMO3 | 8.00E-06 |
| MDM20 | ***M***itochondrial ***D***istribution and ***M***orphology | mito inheritance | C12orf30 | 5.00E-13 |
| MDM35 | ***M***itochondrial ***D***istribution and ***M***orphology | mito, cyto, nuc | TRIAP1 | 2.00E-08 |
| MET7 | ***MET***hionine requiring: mito DNA maintenance | cyto, mito | FPGS | 2.00E-82 |
| MIP1 | ***MI***tochondrial DNA ***P***olymerase:maintenance of mitochondrial genome | mito | POLG | 1.00E-111 |
| MSD1 | ***M***itochondrial aminoacyl-tRNA ***S***ynthetase, Aspartate (***D***) | mito | DARS2 | 4.00E-82 |
| MSE1 | ***M***itochondrial aminoacyl-tRNA ***S***ynthetase, Glutamate (***E***) | mito | EARS2 | 4.00E-77 |
| MSM1 | ***M***itochondrial aminoacyl-tRNA ***S***ynthetase, ***M***ethionine | mito | MARS2 | 2.00E-84 |
| MSY1 | ***M***itochondrial aminoacyl-tRNA ***S***ynthetase, tyrosine (***Y***) | mito | YARS2 | 7.00E-68 |
| NEW1 | ATP binding cassette family member | cyto, mito | GCN20 | 2.00E-45 |
| NSR1 | Nucleolar protein, pre-rRNA processing | mito, nuc, nucleolus | NCL | 7.00E-34 |
| ORF | ORF, Uncharacterized transcription factor? | cyto, mito | ANKZF1 | 5.00E-29 |
| PSK2 | ***P***as domain-containing ***S***erine/threonine ***K***inase | cyto, mito | PASK | 3.00E-47 |
| RAD50 | ***RAD***iation sensitive: Subunit of MRX complex | nuc, mito | RAD50 | 4.00E-158 |
| SAC1 | ***S***uppressor of ***AC***tin | ER, golgi, mito | SACM1L | 1.00E-95 |
| SIN3 | ***S***witch ***IN***dependent | mito | SIN3A | 3.00E-96 |
| SOD1 | ***S***uper***O***xide ***D***ismutase | mito, cytosol | SOD1 | 3.00E-43 |
| SPT7 | ***S***u***P***pressor of ***T***y | nuc, mito | BAZ1A | 3.00E-12 |
| SUV3 | ***SU***ppressor of ***V***ar1: RNA helicase | mito | SUPV3L1 | 8.00E-80 |
| TCM62 | ***T***ri***C***hoder***M***in resistance | mito | HSPD1 | 8.00E-14 |
| ULS1 | ***U***biquitin ***L***igase for ***S***UMO conjugates: | nuc, mito | HLTF | 1.00E-61 |

**^1^** Functional assignment determined from Saccharomyces genome database (SGD).

**^2^** Cellular component determined from SGD; mito: mitochondrion; cyto: cytoplasm; prot: proteasome; memb: membrane; ER: endoplasmic reticulum; nuc: nucleus

**^3^** Human ortholog identified by BLAST analysis of yeast protein sequences (obtained at SGD) against the human RefProtein sequences database at NCBI.
