# Supplementary material for: Comparative Genome-Wide Screening Identifies a Conserved Doxorubicin Repair Network That Is Diploid Specific in Saccharomyces cerevisiae
Source: PLoS One. 2009 Jun 8;4(6):e5830. doi: 10.1371/journal.pone.0005830 (PMC2688081; doi:10.1371/journal.pone.0005830)
Supplement: Table S4 — Yeast doxorubicin resistance genes whose protein products are orthologs of human proteins encoded by genes mutated in breast cancer. Table listing yeast doxorubicin resistance proteins that are orthologs of human proteins encoded by genes found to be mutated in breast cancer (see text Discussion section for complete description). (0.08 MB DOC) [file pone.0005830.s004.doc]

Table S4. Yeast doxorubicin resistance genes whose protein products are orthologs of human proteins encoded by genes mutated in breast cancer.

| **DOX^R^ yeast protein^1^** | **Diploid *MAT***  **expression^2^** | **P-value** | **Mutated in breast cancer human ortholog^3^** | **Yeast protein location^4^** | **Yeast function^5^** |
| --- | --- | --- | --- | --- | --- |
| Rkr1 | No | 6.00E-03 | AMFR | nucleus, cyto | chromatin modification |
| Slx8 | No | 3.00E-03 | BRCA1 | nucleus, cyto | ubiquitin ligase complex |
| Ris1 | No | 5.00E-18 | CHD5 | mito, nucleus | silencing |
| Dhh1 | No | 4.00E-49 | DDX10 | cytoplasm | mRNA decay at P-body |
| Akr1 | No | 3.00E-04 | CENTB1 | membrane | phermone response |
| Akr1 | No | 1.00E-12 | EHMT1 | membrane | pheremone response |
| Set2 | No | 3.00E-15 | EHMT1 | nucleus | histone methyltransferase |
| Yar1 | No | 5.00E-04 | EHMT1 | cytoplasm | oxidative stress |
| Tup1 | No | 7.00E-19 | FLJ10458 | nucleus | transcription repressor |
| Tup1 | No | 3.00E-12 | RNU3IP2 | nucleus | transcription repressor |
| Asc1 | No | 1.00E-16 | FLJ10458 | cytoplasm | ribosome small subunit |
| Mig1 | No | 4.00E-08 | FLJ13479 | cytoplasm, nucleus | transcription factor |
| Sfp1 | No | 1.00E-04 | FLJ13479 | cytoplasm | damage response |
| Sfp1 | No | 2.00E-04 | GLI1 | cytoplasm | damage response |
| Mig1 | No | 1.00E-09 | GLI1 | cytoplasm, nucleus | transcription factor |
| Mig1 | No | 5.00E-10 | ZFP64 | cytoplasm nucleus | transcription factor |
| Snf5 | Yes | 9.00E-03 | ABCA3 | nucleus | chromatin remodelling |
| Snf5 | Yes | 1.00E-06 | NCOA6 | nucleus | chromatin remodelling |
| Pho2 | No | 5.00E-05 | HOXA3 | nucleus | transcription factor |
| **Gin4** | **No** | **3.00E-55** | **KIAA0999** | **bud neck** | **kinase bud growth** |
| **Bck1** | **No** | **3.00E-39** | **MAP3K6^6^** | **intracellular** | **PKC signaling pathway** |
| Fab1 | No | 8.00E-10 | MTMR3 | mitochondrion | vacuolar membrane kinase |
| Fab1 | No | 1.00E-07 | ZFYVE26 | mitochondrion | vacuolar kinase |
| Nhx1 | no | 2.00E-37 | SLC9A2 | late endosome | osmotolerance |
| Ptc1 | No | 2.00E-28 | PPM1E | cytoplasm, nucleus | mitochondrial inheritance |
| Rad50 | No | 3.00E-06 | KTN1 | nucleus mitochondrion | DSB repair |
| Sac6 | No | 1.00E-07 | FLNB | actin cytoskeleton | actin bundling function |
| Sac7 | No | 2.00E-03 | STARD8 | intracellular | signaling to the actin cytoskeleton |
| **Sky1** | **No** | **1.00E-17** | **PRPF4B** | **cytoplasm** | **reg mRNA metabolism proteins** |
| **Slt2** | **No** | **6.00E-26** | **PRPF4B** | **cyto nucleus bud tip** | **map kinase** |

^1^ Diploid yeast deletions identified in a genome-wide screen for hypersensitivity and/or slow growth in response to Dox (n = 376).

^2^ Each Dox^S^ diploid deletion strain was tested for mating ability as described in Fig. 2C. Only the *snf5Δ* strain demonstrated aberrant mating ability as a diploid suggesting that the defect in Dox resistance may be in an indirect repair pathway regulated by *MAT*.

^3^ The list of candidate genes found to be mutated in breast cancer (n = 122; [106]) were aligned by BLAST analysis against the yeast protein products coded by the genes found to be sensitive to Dox in the genome-wide screen in diploid yeast. P-values smaller than E-03 were considered significant as previously described [40].

^4^ Subcellular protein localization as listed in SGD.

^5^ Yeast gene functions as listed in SGD.

^6^ The MAP3K6 gene and “activation of JNK activity” was identified as significantly overrepresented in breast cancer. This association was derived from the list of mutated breast cancer genes described above [106].
